# Supplementary material for: Barriers and facilitators to uptake and retention of inner-city ethnically diverse women in a postnatal weight management intervention: a mixed-methods process evaluation within a feasibility trial in England
Source: BMJ Open. 2020 Jul 21;10(7):e034747. doi: 10.1136/bmjopen-2019-034747 (PMC7375430; doi:10.1136/bmjopen-2019-034747)
Supplement: Author's manuscript [file bmjopen-2019-034747.draft_revisions.pdf]

BMJ Open is committed to open peer review. As part of this commitment we make the peer review history of every article we publish publicly available.

When an article is published we post the peer reviewers' comments and the authors' responses online. We also post the versions of the paper that were used during peer review. These are the versions that the peer review comments apply to.

The versions of the paper that follow are the versions that were submitted during the peer review process. They are not the versions of record or the final published versions. They should not be cited or distributed as the published version of this manuscript.

BMJ Open is an open access journal and the full, final, typeset and author-corrected version of record of the manuscript is available on our site with no access controls, subscription charges or pay-per-view fees (http://bmjopen.bmj.com).

If you have any questions on BMJ Open's open peer review process please email info.bmjopen@bmj.com

BMJ Open

Barriers and facilitators to uptake and retention in a postnatal weight management intervention: application of the COM-B model

Journal:	*BMJ Open*
Manuscript ID	bmjopen-2019-034747
Article Type:	Original research
Date Submitted by the Author:	03-Oct-2019
Complete List of Authors:	Taylor, Cath; University of Surrey, School of Health Sciences<br>Bhavnani, Vanita; National Childbirth Trust<br>Zasada, Magdelena; University of Surrey, School of Health Sciences<br>Ussher, Michael; St George's University of London, Division of Population Health Sciences and Education<br>Bick, Debra; University of Warwick, Warwick Clinical Trials Unit, Warwick Medical School
Keywords:	Maternal medicine < OBSTETRICS, PUBLIC HEALTH, QUALITATIVE RESEARCH

SCHOLARONE™
Manuscripts

BMJ

I, the Submitting Author has the right to grant and does grant on behalf of all authors of the Work (as defined in the below author licence), an exclusive licence and/or a non-exclusive licence for contributions from authors who are: i) UK Crown employees; ii) where BMJ has agreed a CC-BY licence shall apply, and/or iii) in accordance with the terms applicable for US Federal Government officers or employees acting as part of their official duties; on a worldwide, perpetual, irrevocable, royalty-free basis to BMJ Publishing Group Ltd ("BMJ") its licensees and where the relevant Journal is co-owned by BMJ to the co-owners of the Journal, to publish the Work in this journal and any other BMJ products and to exploit all rights, as set out in our licence.

The Submitting Author accepts and understands that any supply made under these terms is made by BMJ to the Submitting Author unless you are acting as an employee on behalf of your employer or a postgraduate student of an affiliated institution which is paying any applicable article publishing charge ("APC") for Open Access articles. Where the Submitting Author wishes to make the Work available on an Open Access basis (and intends to pay the relevant APC), the terms of reuse of such Open Access shall be governed by a Creative Commons licence – details of these licences and which Creative Commons licence will apply to this Work are set out in our licence referred to above.

Other than as permitted in any relevant BMJ Author's Self Archiving Policies, I confirm this Work has not been accepted for publication elsewhere, is not being considered for publication elsewhere and does not duplicate material already published. I confirm all authors consent to publication of this Work and authorise the granting of this licence.

For peer review only

Barriers and facilitators to uptake and retention in a postnatal weight management intervention: application of the COM-B model

Cath Taylor[1]; Vanita Bhavnani[2]; Magdalena Zasada[1]; Michael Ussher[3]; Debra Bick[4] and on behalf of the SWAN trial team

[1] School of Health Sciences, University of Surrey, Guildford UK

[2] National Childbirth Trust, London UK

[3] Division of Population Health and Education, St George's University of London, London UK

[4] Warwick Clinical Trials Unit, Warwick Medical School, University of Warwick, Coventry UK

Corresponding author:

Cath Taylor

School of Health Sciences

University of Surrey

Guildford GU2 7YH

Email: cath.taylor@surrey.ac.uk

Tel: +44 1483 688862

Word Count: 3981

Keywords: postpartum; maternal health; weight management; health behaviour

For peer review only

For peer review only - http://bmjopen.bmj.com/site/about/guidelines.xhtml

Abstract

Objectives: To understand the barriers and facilitators to uptake and retention of postnatal women randomised to a commercial group weight management intervention using the COM-B (Capability, Opportunity, Motivation - Behaviour) behaviour change model.

Design: Concurrent mixed methods (qualitative dominant) process evaluation nested within a feasibility randomised controlled trial, comprising questionnaires and interviews at six and 12 months post-birth.

Setting: One National Health Service maternity unit in an inner city area in the south of England.

Participants: 98 postnatal women with BMIs>25kg/m^2 (overweight/obese) at pregnancy commencement.

Intervention: Twelve week Slimming World® (SW) commercial group weight management programme, commencing anytime from 8 to 16 weeks postnatally.

Primary and secondary outcome measures: Data regarding uptake and retention from questionnaires and interviews conducted six and 12 months post birth, analysed thematically and mapped to the COM-B model.

Results: Barriers to SW uptake mostly concerned opportunity issues (e.g., lack of time or childcare support) though some women also lacked motivation, not feeling that weight reduction was a priority, and a few cited capability issues such as lacking confidence. Weight loss aspirations were also a key factor explaining retention, as were opportunity issues, particularly in relation to the social context of the groups (extent of group identity; relationship with the group consultant) and perceived support from and fit with family lifestyle. In addition, barriers relating to beliefs and expectations about the SW programme were identified, including concerns regarding compatibility with breastfeeding and importance of exercise. Women's understanding of the SW approach, and capability to implement into their lifestyles, appeared related to level of attendance (dose-response effect).

Conclusions: Uptake and retention in commercial weight management programmes may be enhanced by applying behaviour change techniques to address the barriers impacting on women's perceived capability, motivation and opportunity to participate.

Trial registration: This is the process evaluation of a feasibility RCT (ISRCTN 39186148)

For peer review only - http://bmjopen.bmj.com/site/about/guidelines.xhtml

Article summary

Strengths and limitations of this study

- The need to consider postnatal weight management interventions has been highlighted by the lack of benefit shown by pregnancy interventions. This is this first study to use behaviour change theory to explore uptake and retention of postnatal women in a weight management intervention
- The sample were from an ethnically and economically diverse inner-city population
- The intervention is an evidence-based commercially available programme available throughout the UK and in several other countries worldwide
- As a single centre feasibility study, findings cannot necessarily be generalised
- Techniques proposed to mitigate the emergent barriers require further evaluation.

Introduction

The proportion of women entering pregnancy with an overweight or obese Body Mass Index (i.e. BMIs of $\geq 25kg/m^2$) is high and increasing, accounting for approximately half of all pregnancies in the UK, [1] and USA.[2] Pregnant women with obese BMIs have an increased risk of adverse outcomes for themselves (e.g. pre-eclampsia, gestational diabetes) and their infants (e.g. stillbirth, congenital anomalies).[3] Furthermore, retaining weight postnatally is associated with other poor health behaviours including smoking, poor diet, lack of exercise and not breastfeeding.[4-6] It also increases risks for long-term obesity, hypertension, diabetes and degenerative joint disease in women,[7] and their infants are at risk of higher BMI and blood pressure in childhood and young adulthood.[6]

Intervening prior to pregnancy is challenging as most pregnancies are unplanned[8] and women are not generally in touch with health services until they are pregnant. Due to limited evidence of effective weight management interventions during pregnancy[9] attention has shifted to the postnatal period. However, studies of postnatal weight management have methodological limitations (e.g. lacking information on randomisation or response rates), and wide heterogeneity in relation to intervention and evaluation.[10-11] Furthermore, neither uptake nor retention are explicitly reported in these reviews.

In general populations, commercial weight management programmes were more effective than health service delivered programmes,[12] with the advantage of being nationally available and tailored to community needs and settings. Few cater for women in pregnancy or postpartum; one

For peer review only - http://bmjopen.bmj.com/site/about/guidelines.xhtml

exception is Slimming World (SW, www.slimmingworld.co.uk) who have produced guidelines and support in collaboration with the Royal College of Midwifery.

According to the COM-B (capability, opportunity, motivation and behaviour) model,[13] successful behaviour change, such as weight management, requires an individual to be *capable* of performing the behaviour, have the *opportunity* to carry out the behaviour, and be *motivated* to change the behaviour. For general populations, COM-B has been applied in a review of uptake and retention in weight management programmes (including SW).[14] Uptake was greatest in those motivated to attend and this was highly related to capability (knowledge and skills to change behaviour) and opportunity, particularly social opportunity. Social opportunity (including within the programme; e.g. social support, supportive leader, and outside; e.g. family and friends) was the dominant driver of retention, with psychological capability also important (highest retention when there was self-monitoring and feedback on behaviour). A recently completed feasibility randomised controlled trial of access to SW in a postnatal population[15] presented data on recruitment, retention and other feasibility outcomes, which were supportive of progression to a definitive trial. This paper reports results from the mixed methods process evaluation of the study, the objective being to use the COM-B to examine the barriers and facilitators to uptake and retention of women allocated to the intervention.

Methods

Reported according to COREQ guidelines.

Design: Concurrent mixed methods (qualitative dominant) process evaluation nested within a feasibility randomised controlled trial, comprising questionnaires and interviews at six and 12 months post-birth.

Setting: One NHS maternity unit in an ethnically diverse inner city area in the south of England with high levels of socioeconomic disadvantage, mobility and migration.

Participants: Postnatal women aged ≥18 with BMIs>25kg/m^2 at pregnancy commencement or with normal BMIs (18.5kg/m^2-24.9kg/m^2) who met criteria for excessive gestational weight gain (EGWG) at 36 weeks gestation, randomly allocated at 36 weeks gestation to access 12-weekly SW sessions, commenced anytime from 8-16 weeks post-birth (n=98). Full inclusion/exclusion criteria are published elsewhere[15].

Intervention: Slimming World® (Alfreton, UK), is a commercial weight management programme. The programme is underpinned by behaviour change theory and techniques, including social cognitive

For peer review only - http://bmjopen.bmj.com/site/about/guidelines.xhtml

theory[16] with a focus on motivation and self-efficacy for weight management and reducing relapse. Key techniques include goal setting, self-monitoring, social support, and positive reinforcement.[17-18] Consultants leading the groups receive standardised training from SW dieticians and nutritionists, which includes motivational skills to support positive lifestyle changes, nutrition, food facts, and role of exercise and activity in health and weight management. Groups follow a standard format, starting with members being weighed (confidentially), whilst new members attend an introductory session, followed by group session (called Image Therapy) where members discuss their experiences of weight management that week, facilitated by the consultant and aimed at supporting behaviour change, and encouraging peer support. Sessions can include basic cooking skills, taking cost, cultural preferences and time constraints into account. Physical activity is encouraged and rewarded through 'body magic awards', including redefining what 'activity' can include (e.g. housework, gardening). Members also have access to SW online resources (weight tracking, recipes, tips, motivational articles etc). The dietary plan encourages most foods (80%) to be fruit, vegetables, carbohydrates and lean protein; a smaller but 'mandatory' allowance for calcium-rich and fibre-rich foods; and a daily optional allowance for foods high in fat or sugar. Women who are breastfeeding have an increased allowance for calcium-rich foods.

Women allocated to the intervention were offered (fees waived) the standard SW membership (12 sessions over 14 weeks). SW guidance is that in order to achieve a 5% weight loss, a difference considered to improve health outcomes, individuals need to attend at least 10 of 12 sessions.[19] In our process evaluation, we considered whether women stayed for whole group sessions (or left after being weighed), and whether women attended 10+ sessions, 6-9 sessions; 1-5 sessions or did not attend at all. On completion of the 12 weeks, women could continue with standard SW fees applicable. Women received an information leaflet about joining SW, a congratulations text from the Research Midwives on the birth of their baby, and reminder texts at 6-8 weeks postnatally. From 8-16 weeks postnatally they could call SW to register and locate their nearest groups.

Questionnaire component: All women were invited to complete questionnaires at baseline (36 weeks gestation) and six months post-birth. Responders at six-months were invited to complete a further questionnaire at 12 months post-birth. The six and 12 month questionnaires, which included measures of dietary intake, mental and physical health and lifestyle behaviours (reported elsewhere), also assessed attendance at SW. This included uptake (asking if they attended any sessions and, if so, age of their baby when attending their first session); and retention (how many sessions attended in total; if they stayed for the whole session or just got weighed). Two open questions asked why they did not stay for whole sessions (if relevant), and how useful they found

For peer review only - http://bmjopen.bmj.com/site/about/guidelines.xhtml

the sessions. Women that had not attended any sessions were asked to explain why they chose not to attend.

Interview component: Interviews were conducted by a female experienced qualitative researcher (VB) with a purposively selected sample of women at approximately six months post-birth and 12 months post-birth. Criteria for inviting women captured diversity (in line with guidance[20]) in relation to: attendance at SW sessions (women who attended 10+ sessions vs. attended fewer or did not attend at all); weight change; and ethnicity). The same criteria were applied at 12 months post-birth, but also included six women from the initial sample to provide a nested longitudinal sample. The topic guide included: motivation for study participation, experiences of the intervention, and reasons for uptake and retention (or not), with prompts informed by the COM-B model. Women were approached initially by Research Midwives who provided a study information sheet. With consent, their contact details were shared with the researcher who contacted them to answer questions, confirm consent and arrange interviews. All interviews were by telephone (face-to-face was offered), and lasted between 20-50 minutes (average 45 minutes). Interviews were-audio recorded.

Analysis:

Quantitative questionnaire data were analysed descriptively. Analysis of open questions and interview data was underpinned by the COM-B model[13] and used the Framework method[21]. Interviews were transcribed verbatim. Transcripts were read by two researchers (CT & MZ for questionnaires; CT & VB for interviews), who independently noted key themes. Coding was compared and discussed before final coding and labelling. Data were examined to identify themes in relation to capability, motivation and opportunity factors (e.g. comparing women that attended different 'doses' of sessions; and those that lost or gained weight). Data were examined longitudinally if women completed both questionnaires/interviews. As the COM-B components are not mutually exclusive, data often fit several dimensions.

Integration of findings: Data from questionnaires and interviews were analysed separately before being compared, and integrated into a single framework. Key barriers to uptake and retention were mapped to behaviour change techniques for enhancing uptake and retention.

Quotations are anonymised with pseudonyms (flowers). Each quote is labelled with the pseudonym, number of sessions attended, and weight change from baseline to the time point they provided data.

For peer review only - http://bmjopen.bmj.com/site/about/guidelines.xhtml

Patient and Public Involvement:

The choice of SW as the intervention was heavily influenced by feedback from local women who had participated in a trial of weight management during pregnancy.[22] Their views included that the intervention should be non-NHS, peer-supported to enable social support but not just 'new mums' (though knowledgeable of their needs), flexible, and suitable for taking their babies. Four women who had recently given birth and experienced weight management issues were recruited to join our PPI group via advertisements in community midwifery locations. They were supported by a researcher (VB) to contribute to the development of study materials, conduct of the trial, interpretation and dissemination of findings.

Ethics approval was granted by the Health Research Authority London – Camberwell St Giles REC (16/LO/1422).

Results

Of 98 women randomised to the intervention, 83 (85%) completed the six month questionnaire, 69 of whom (69/83, 83%) also completed the 12 month questionnaire. At least one of the two questions about SW was answered by all women who attended at least one SW session. Fifty-two (53%) women did not attend any sessions and 39 (75%) gave a reason for not attending.

Interviews were conducted with 13 women immediately post-intervention (approximately six to eight months post-birth); and with nine women (six of whom had participated at six-months) at 12 months post-birth. The characteristics of participants are provided in Table 1.

Uptake

Just over half of the intervention women did not attend any SW sessions. Most commonly this related to 'opportunity', particularly lack of time to attend. Some had difficulties organising childcare or fitting SW sessions into their family schedule, for example sessions clashing with older children's bedtimes. One woman reported breastfeeding on demand as a limiting factor, either not knowing she could take her baby with her (or not choosing to do so):

For peer review only - http://bmjopen.bmj.com/site/about/guidelines.xhtml

> *"I chose to breastfeed on demand so I would not have been able to attend any scheduled sessions easily."* [Rose; gained 12kg 12mth]

Two participants found the offer too soon post-birth; stating that six months postpartum would have been easier. Other 'opportunity' factors included their own or their infants ill health, or social factors including lack of confidence (capability) due to perceived language barriers reported by a non-native English speaker, and others stating they were leaving the country for an extended period, or leaving London and not realising that they could attend groups elsewhere. Two participants experienced errors in registration that prevented them from enrolling in time. The location of the groups was only mentioned as a barrier by five women.

In addition to these 'opportunity' factors, there were 'motivational' barriers to uptake for some women who were either happy with their weight, stated they had already lost weight, or did not feel motivated to lose weight. Others did not believe SW to be suitable, either because they did not believe it promoted an effective or balanced diet, or due to a perceived lack of relevance for new mums:

> *"I was asked whether I would be comfortable with people cheering if I'd lost weight which made me think I had done something wrong rather than have a child! And also that the programme was targeted to teach me how to eat better- e.g. by using olive oil and not eating ready meals - which I'd never done. So it sounded more like something for unhealthy people with bad habits, not new mums."* [Lily; lost 6.5kg 12mth]

Retention

Although nearly half of women assigned to SW attended at least one session (46/98, 47%), only half of these (n=19/46) attended the full 'dose' (i.e. 10+ sessions); the mean number of sessions was 6.74 (SD 3.94). Five key themes emerged which differed across the three groups (10+, 6-9 and 1-5 sessions) and help to explain barriers and facilitators to retention. Each is considered below and summarized in Table 2 where they are mapped to behaviour change techniques (BCTs) that may mitigate barriers.

Motivation: weight loss aspirations

Women who attended 10+ sessions (and some women who attended fewer sessions but lost weight over the data collection period) tended to perceive themselves as overweight, to understand why they gained weight, and to perceive weight loss as a priority.

For peer review only - http://bmjopen.bmj.com/site/about/guidelines.xhtml

> *"I guess I got into the routine of comfort eating and putting on more weight I thought that if I didn't take this opportunity I would have really been at a loss."* [Violet; 11; lost 17.5kg 12mth]

In contrast, women who attended 6-9 sessions (and gained weight) – although acknowledging they were overweight and understanding why – were less likely to prioritise weight loss, most commonly citing that adapting to life with their baby was more important:

> *"For me three months into having had a baby, I wasn't standing there thinking I've got to lose the weight. I was standing there thinking, wow how do I reacclimatise and recalibrate my life to accommodate this little person."* [Aster; 6; gained 2.8kg 12mth]

Weight reduction was also given low priority by women who attended 1-5 sessions, who were less likely to recognise they were overweight, and/or state they did not gain much weight during their pregnancy, as the rationale for not prioritising weight reduction.

Motivation (psychological capability): Beliefs and expectations regarding weight loss

A common theme among women attending 10+ sessions was that the intervention had altered their beliefs about how to lose weight, particularly regarding the role of exercise:

> *"I've gone on various diets but half-heartedly, and I've always done it though exercise … So it's a kind of revelation that it's 90% food and 10% exercise which is what I learnt at Slimming World … seeing 5½ lbs loss in the first week confirmed it would work. I am proud of myself."* [Heather; 12; lost 2.6kg 12mth]

Women who attended fewer SW sessions were more likely to question the intervention, often because they felt it failed to prioritise exercise as they had expected.

> *"I just felt there should also be an activity, there wasn't any exercise classes or anything like that and maybe that side of things didn't attract me"* [Iris; 1; gained 4.4kg 12mth]

Other beliefs that explained poor retention included that SW was unsuitable for breastfeeding mothers, or that their needs were broader than weight management (e.g. also requiring postnatal mental-wellbeing support).

Motivation/Capability: Understanding and implementing the intervention

A key 'capability' factor was the extent to which women understood and were able to implement the SW programme. Women who attended 10+ sessions described following the dietary plan and using SW online resources to support them. They were motivated by the health and wellbeing benefits they experienced for themselves and for their families:

For peer review only - http://bmjopen.bmj.com/site/about/guidelines.xhtml

"It felt to me to be sustainable, not a harsh diet that I was only going to do for a few weeks. I was still enjoying myself and enjoying my food and I was still losing weight, so that was very motivating." [Hibiscus; 10; gained 0.3kg 12mth]

In contrast, women who attended 6-9 sessions appeared to have a poorer understanding of the SW plan and/or reported difficulties adapting to the plan:

"It's quite a low sugar, low fat diet and I was reluctant to cut my fat levels too much so I tried to keep some full fat milk and I would still eat more fatty avocados. I just took the free food and went with it without actually counting syns…I am not convinced that sticking to the plan would actually help me maintain longer term." [Lavender; 9; lost 0.4kg 12mth]

Many gained weight and their motivation to attend may have been affected by lack of perceived benefit. For women who attended 1-5 sessions these barriers were most apparent, suggesting that there might be a 'dose-response' effect: the more sessions women attended, the more likely it was that they would feel 'capable' and thereby 'motivated' to successfully implement lifestyle changes.

Social opportunity and Motivation: Social context

Opportunity factors relating to both the internal (intervention) and external (home support) social context were important explanatory factors for retention. Internally, women who attended 10+ sessions were most likely to describe identifying with other SW group members, seeing their group consultants as helpful in adapting the programme to their circumstances (e.g. breastfeeding) and generally perceived the groups as a supportive and safe environment:

"I was positively surprised how much the group was supportive and I'm also new in the area so it was a really good way to meet people, like meet new mums. I keep bumping into members in the supermarket and in the park exercising …. I felt part of a little community" [Orchid; 10; lost 3.7kg 12mth]

"*Consultant very positive, made baby welcome and was supportive*" [Peony; lost 3.1kg 12mth]

Those who attended <10 sessions were more likely to express negative views about their groups or consultants. Several mentioned having difficulty bonding or socially identifying with other group members, or that their consultants were unsympathetic to their specific postnatal challenges:

"*The atmosphere was very city/work based and not welcoming to new mothers*" [Poinsettia; 1; gained 1.8kg 6mth]

For peer review only - http://bmjopen.bmj.com/site/about/guidelines.xhtml

"The woman who ran the group, wasn't very nice, she was very critical…" [Hyacinth; 7; gained 13.7kg 12mth]

In relation to 'external' factors, women attending 10+ sessions commonly reported benefitting from support from their partners, either practically (e.g. childcare whilst they attended), or willingness to adapt their diets alongside their partner. One husband also joined SW: *"I took my husband with me. He lost quite a lot of weight as well. So it was good."* [Orchid; 10; lost 3.7kg 12mth]

For others, the lack of partners' support presented a barrier to participating and/or losing weight:

"It's a lot easier to do [follow the plan] *on the nights he* [husband] *is working. I suppose he just likes certain food … he likes to have a proper meal."* [Hibiscus; 10; gained 0.3kg 12mth]

"Husband not supportive" [Poinsettia; 1; gained 1.8kg 6mth]

Physical opportunity: Accessibility of the intervention

A key opportunity factor was the accessibility of the intervention, which either facilitated or created a barrier to attendance. Issues included *timing of onset* of the intervention, *ease of attendance* and *duration of the intervention*. Women had diverse views that were unrelated to the number of sessions attended or their weight change. In relation to intervention onset, most reported that starting SW between 8-16 weeks post-birth was acceptable. However some felt it was too early in the postnatal period, coinciding with the time they might be experiencing difficulties with breastfeeding, concerns over their baby's health and the general transition to parenthood:

"*couldn't concentrate while had baby with me as breastfeeding … offer too early in postnatal period, bad timing of groups with baby, exhausted*" [Primrose; 2; lost 0.7kg 12mth]

Regarding ease of attendance, most women could access a SW group that was located and timed to suit them. However, two women who attended <10 sessions, stated having difficulties in this regard:

"That [group] *was the only one within walking distance from my house…On most days; I didn't stay* [after being weighed]*...because of the inconvenient timing she was by that point in time she would be really ratty and ready for sleep."* [Hyacinth; 7; gained 13.7kg 12mth]

For peer review only - http://bmjopen.bmj.com/site/about/guidelines.xhtml

Other 'opportunity' reasons for missing sessions and/or not completing the programme included holiday plans, illness (in woman or infant), and childcare difficulties (for siblings or where they didn't want to take their babies with them).

The third key accessibility issue concerned the intervention duration. Many who completed the programme considered it to be too short:

> *"I don't think 12 weeks is long enough to see any real benefits and to establish healthy eating/lose weight long term. I would say 6 months is optimal"* [Tulip; 6; gained 3.6kg 12mth].

Women could continue beyond 12 weeks but fees were applicable. Some women did this, for others the timing of the intervention ceasing presented a major barrier as coincided with the end of maternity benefit:

> *"Once the 12 weeks were up … that was the time when I [had] no money, so £5 a week ended up being quite a lot of money that I couldn't really afford … if it was possible to do it for a year [during] maternity leave that would be fantastic."* Azalea; 11; lost -1.8kg 12mth]

Discussion

This is the first study to use behaviour change theory to explore uptake and retention in a postnatal weight management intervention. A number of modifiable barriers to uptake and retention were identified. Fewer than half of the intervention arm women attended at least one session; a rate consistent with weight management studies in pregnancy[23-24] and general population.[25] Reported barriers could potentially be addressed using BCTs (see Table 2) - for example through strengthening motivation to lose weight by tackling incompatible beliefs and/or supporting consideration of the pros and cons of weight loss and providing evidence regarding health consequences. Previous studies of weight management in pregnancy have highlighted the difficulty that midwives and other health professionals have discussing weight with women,[26-28] and indicate a training need to support effective conversations. Some women also found the timing of the intervention challenging: offering a wider commencement window (e.g. allowing women to start SW anytime up to six months post-birth) may have increased uptake.

Only 19% of intervention arm women received the 'full dose' of 10+ sessions. Retention (and uptake) may be enhanced by offering enhanced support to help women adapt the dietary plan to their lifestyle and family needs, to address capability issues (e.g. understanding and/or implementing the diet); and opportunity factors might be mitigated by ensuring women know there is flexibility regarding the group they attend; and by encouraging women to identify those in their social environment who will

For peer review only - http://bmjopen.bmj.com/site/about/guidelines.xhtml

support their dietary changes and weight loss aspirations. There appeared to be a ‘dose-response’ relationship with women attending 10+ sessions reporting the greatest benefit. This concurs with findings from a general population study where those attending at least 75% of the offered sessions (also provided by SW) achieved the greatest weight loss.[19] Women who did not receive the full dose often had erroneous perceptions about the programme and its effectiveness or compatibility with their postnatal status, which in turn reinforced their decision to give up. The use of techniques to shape knowledge regarding effective weight loss and the SW programme at the time of study recruitment may mitigate this barrier and more closely align their expectations with the programme. As well as widening the commencement window, findings suggest that a longer duration of intervention may improve the sustainability of weight loss, particularly as the current duration ended at a similar time to women’s statutory maternity leave payments ending. Recent evidence from an RCT comparing weight loss in adults with obese BMIs allocated to brief (booklet only), 12 week or 52 week programmes found the 52 week programme was both more clinically and cost effective than shorter programmes.[29]

To our knowledge this is the first study that has used behaviour change theory to understand uptake and retention of postnatal women in a weight management programme, and included a diverse inner-city population with women with various levels of attendance. The use of framework analysis ensured data analysis was transparent and enabled all research team members to contribute. As a single centre feasibility study, findings cannot be generalised, though the intervention is commercially available throughout the UK and in several countries worldwide, which may enhance the relevance of findings. Findings will be used to optimise the potency of the intervention in a future trial, with an embedded process evaluation to further evaluate the impact of proposed BCTs on uptake and retention.

Author contributions

CT and DB developed the study concept and study design, with the support of MU. VB completed data collection and VB, CT and MZ were responsible for data analysis. All authors contributed to interpretation of findings. DB had primary responsibility for overseeing the overall SWAN trial. CT drafted the first version of the manuscript, with the support of MU, MZ and DB. All authors read and approved the final manuscript.

SWAN trial team members:

For peer review only - http://bmjopen.bmj.com/site/about/guidelines.xhtml

Dr Amanda Avery, University of Nottingham

Dr Andy Healey, King's College London

Ms Nina Khazaededah, Guy's and St Thomas' NHS Foundation Trust

Dr Sara McMullen, National Childbirth Trust

Professor Eugene Oteng-Ntim, King's College London

Dr Bimpe Oki, Lambeth and Southwark Public Health

Professor Lucilla Poston, King's College London

Mr Paul Seed, King's College London

Acknowledgements:

We thank the women who participated in this study, and Sheila O'Connor and Victoria Craig (research midwives) for supporting recruitment and data collection. We would also like to thank members of our trial steering group.

Funding:

This study is funded by the National Institute for Health Research (NIHR) Public Health Research Programme (ref no. 14/67/14). The views expressed are those of the author(s) and not necessarily those of the NIHR or the Department of Health and Social Care.

Competing interests:

AA (a member of the SWAN trial team), alongside her academic position at the University of Nottingham, also holds a consultancy position at Slimming World®(Alfreton, UK). Neither AA nor Slimming World® (Alfreton, UK) had access to the study data or were involved in data collection or analyses. DB was supported by the National Institute for Health Research (NIHR) Collaboration for Leadership in Applied Health Research and Care South London (NIHR CLAHRC South London) at King's College Hospital NHS Foundation Trust. The other authors declare that they have no competing interests in relation to this study.

Data Sharing Statement:

The data sets generated and/or analysed as part of this study are not publicly available due to them containing information that could compromise research participant privacy/consent but are available from the corresponding author on reasonable request.

References

1. NHS Maternity Statistics 2017-18 https://files.digital.nhs.uk/C3/47466E/hosp-epis-stat-mat-summary-report%202017-18.pdf Last accessed: October 2019
2. Dudenhausen JW 2015 Prepregnancy body weight and gestational weight gain. *Am J Obstet Gynecol* 2015;591-92
3. Leddy MA, Power ML, Schulkin J The Impact of Maternal Obesity on Maternal and Fetal Health. *Rev Obstet Gynecol* 2008;1(4):170-178
4. Hilson JA, Rasmussen KM, Kjolhede CL. Excessive weight gain during pregnancy is associated with earlier termination of breast-feeding among white women. *The Journal of Nutrition* 2006;136(1):140–146.

For peer review only - http://bmjopen.bmj.com/site/about/guidelines.xhtml

5. Vinter CA, Jensen DM, Ovesen P, et al. Postpartum weight retention and breastfeeding among obese women from the randomized controlled Lifestyle in Pregnancy (LiP) trial. *Acta Obstet Gynecol Scand* 2014;93(8):794-801
6. Poston L, Harthoorn LF, van der Beek EM: Obesity in pregnancy: implications for the mother and lifelong health of the child. A consensus statement. *Pediatr Res* 2011;69:175–180.
7. Rooney BL, Schauberger CW, Mathiason MA. Impact of perinatal weight change on long-term obesity and obesity-related illnesses. *Obstet Gynecol* 2005;106:1349–56.
8. Hanson M, Godfrey K, Poston L, et al Pre-conception Health. In: Davies SC Annual Report of the Chief Medical Officer, 2014, The health of the 51%: women. London: Department of Health (2015)
9. Poston L. Obesity in pregnancy: could lifestyle interventions work? *BMC Medicine* 2014;12:201
10. Amorim Adegboye AR, Linne YM. Diet or exercise, or both, for weight reduction in women after childbirth. *Cochrane Database of Systematic Reviews* 2013, Issue 7. Art. No.: CD005627. DOI: 10.1002/14651858.CD005627.pub3.
11. van der Pligt P, Willcox J, Hesketh KD, et al. Systematic review of lifestyle interventions to limit postpartum weight retention: implications for future opportunities to prevent maternal overweight and obesity following childbirth. *Obesity Reviews* 2013;14:792–805 doi: 10.1111/obr.12053
12. Jolly K, Lewis A, Beach J, et al Comparison of range of commercial or primary care led weight reduction programmes with minimal intervention control for weight loss in obesity: Lighten Up randomised controlled trial. *BMJ* 2011;343:d6500 doi: 10.1136/bmj.d6500
13. Michie S, van Strelen M, West R. The behaviour change wheel: A new method for characterising and designing behaviour change interventions. *Implementation Science* 2011;6:42. doi: 10 1186/1748-5908-6-42.
14. Gidlow C, Povey R, Ellis N, et al Uptake and retention in group-based weight management services. Literature review and behavioural analysis. Final report 2018. Public Health England: London. 2018.
15. NIHR Project Page
16. Bandura, A *Social foundations of thought and action: A social cognitive theory*. Prentice-Hall, Inc. 1986.
17. Elfhag K, Rössner S: Who succeeds in maintaining weight loss? A conceptual review of factors associated with weight loss maintenance and weight regain. *Obes Rev* 2005;6(1):67–85.
18. Wing RR, Jeffery RW: Benefits of recruiting participants with friends and increasing social support for weight loss and maintenance. *J Consult Clin Psychol* 1999;67(1):132–138
19. Stubbs RJ, Morris L, Pallister C, et al Weight outcomes audit in 1.3 million adults during their first 3 months' attendance in a commercial weight management programme *BMC Public Health* 2015;15:882
20. O'Cathain A, Hoddinott P, Lewin S, et al. Maximising the impact of qualitative research in feasibility studies for randomised controlled trials: guidance for researchers *Pilot and Feasibility Studies* 2015;1:32
21. Ritchie J, Lewis J: Qualitative research practice: a guide for social science students and researchers. London: Sage 2003
22. Poston L, Bell R, Croker H, et al on behalf of the UPBEAT Trial Consortium. Effect of a behavioural intervention in obese pregnant women (the UPBEAT study): a multicentre, randomised controlled trial. *Lancet Diabetes Endocrinol* 2015;3(10):767–777
23. Davis, DL, Raymond, JE, Clements, V, et al Addressing obesity in pregnancy: The design and feasibility of an innovative intervention in NSW, Australia. *Women Birth* 2012;25:174– 80.

For peer review only - http://bmjopen.bmj.com/site/about/guidelines.xhtml

24. Knight, BA, Wyatt, K. Barriers encountered when recruiting obese pregnant women to a dietary intervention. *Nursing Times*. 2010;106:20– 2
25. Blane DN, McLoone P,Morrison D, *et al*. Patient and practice characteristics predicting attendance and completion at a specialist weight management service in the UK: a cross sectional study. *BMJ Open* 2017;7:e018286. doi:10.1136/bmjopen-2017-018286
26. Holton S, East C, Fisher J. Weight management during pregnancy: a qualitative study of women's and care providers' experiences and perspectives *BMC Pregnancy and Childbirth* 2017;17:351
27. Olander EK, Atkinson L, Edmunds JK, et al The views of pre- and post-natal women and health professionals regarding gestational weight gain: An exploratory study *Sexual & Reproductive Healthcare* 2011;2:43–48
28. Flannery C, McHugh S, Kenny LC, *et al*. Exploring obstetricians', midwives' and general practitioners' approach to weight management in pregnant women with a BMI ≥25 kg/m2: a qualitative study. *BMJ Open* 2019;9:e024808. doi:10.1136/bmjopen-2018-024808
29. Ahern AL, Wheeler GM, Aveyard P, et al Extended and standard duration weight-loss programme referrals for adults in primary care (WRAP): a randomised controlled trial *Lancet* 2017;389:2214–25

For peer review only

For peer review only - http://bmjopen.bmj.com/site/about/guidelines.xhtml

Table 1: Characteristics of study participants

Characteristics	Intervention group n=98	Survey response n=83*	Interview 6 months n=13	Interview 12 months n=9
**Ethnicity**				
White	49 (50%)	43 (52%)	10 (77%)	6 (67%)
Black	35 (36%)	27 (33%)	3 (23%)	3 (33%)
Asian	8 (8%)	8 (10%)	0 (0%)	0 (0%)
Other	6 (6%)	3 (4%)	0 (0%)	0 (0%)
Unknown	0 (0%)	2 (2%)	0 (0%)	0 (0%)
**Age**				
20-24	7 (7%)	4 (5%)	1 (8%)	0 (0%)
25-29	19 (19%)	17 (20%)	3 (23%)	1 (11%)
30-34	33 (34%)	26 (31%)	4 (31%)	3 (33%)
35-39	33 (34%)	30 (36%)	4 (31%)	4 (44%)
40-44	6 (6%)	6 (7%)	1 (8%)	1 (11%)
**BMI at booking**				
<25 with EGWG**	3 (3%)	3 (4%)	0 (0%)	1 (11%)
25-29.9	55 (56%)	51 (61%)	9 (69%)	6 (67%)
>=30	40 (41%)	29 (35%)	4 (31%)	2 (22%)
**Parity**				
Primigravida	50 (51%)	43 (52%)	9 (69%)	5 (56%)
Multigravida	46(47%)	38 (46%)	3 (23%)	4 (44%)
Unknown	2 (2%)	2 (2%)	1 (8%)	0 (0%)
**Number of sessions attended (data from SW)**				
10+	19 (19%)	19 (23%)	6 (46%)	4 (44%)
6 to 9	8 (8%)	8 (10%)	3 (23%)	1 (11%)
1 to 5	19 (19%)	15 (18%)	3 (23%)	3 (33%)
None	52 (53%)	41 (49%)	1 (8%)	1 (11%)

*includes those who responded either to six month questionnaire only, or to both six and 12 month questionnaires

** EGWG Excessive Gestational Weight Gain

For peer review only

For peer review only - http://bmjopen.bmj.com/site/about/guidelines.xhtml

Table 2: Barriers to uptake and retention mapped to COM-B and to potential behaviour change techniques

COM-B	Theme	10+ Slimming World sessions	<10 Slimming World sessions	Behaviour change techniques that could help mitigate barriers
**Motivation**	weight loss aspirations	• self-perceived as overweight<br>• understood why gained weight<br>• desired to lose weight after birth	• some acknowledged overweight and understood why but viewed weight management as less of a priority<br>• Others did not self-perceive as overweight	Provide information (leaflet/brief consultation) at outset to help influence knowledge and understanding regarding weight loss and consequences of action/no action.<br>• Identity (Framing/reframing; Incompatible beliefs; identity associated with changed behaviour)<br>• Comparison of outcomes (Pros and cons; credible source; comparative imagining of future outcomes)<br>• Goal setting (problem solving)<br>• Natural consequences (Information about health consequences; Salience of consequences; Information about social and environmental consequences
**Capability/ Motivation**	beliefs and expectations about SW	• SW and resulting weight loss helped change any pre-existing ideas about how to lose weight, esp re: role of exercise	• Mistrustful of SW, in part as perceived as failing to prioritise exercise in line with pre-existing beliefs, seen as unsuitable for breastfeeding women, or felt gap in care e.g in relation to postnatal mental wellbeing	Leaflet/brief consultation (as above) to include:<br>• Shaping knowledge (Information about antecedents e.g. provide reliable info regarding weight gain/loss; Re-attribution e.g. elicit perceived causes of weight gain/how to lose weight and suggest alternatives)<br>• Goals and Planning (goal setting: behaviour e.g. to make clear at the outset that the SW programme considers the role of exercise but focusses on diet; that 10+ sessions are needed for it to be most effective; Goal setting: outcome – make clear at the outset that the outcome goals are focussed on weight management, but that weight loss may have other positive outcomes such as improved sense of wellbeing/mental health.

				• Comparison of Outcomes (Pros and cons: discuss pros and cons of exercise vs diet for weight loss)
**Capability**	understanding and implementing SW	• Understood dietary plan, found easy to follow, planned meals and used online resources.<br>• Considered plan unrestrictive, sustainable and compatible with postnatal lifestyle<br>• Positive benefits for them and their families	• Did not appear to understand the dietary plan, only partial implementation, and experienced difficulties making dietary adjustments.	Development of a bespoke booklet to give to women at randomisation that summarizes key elements of the programme (e.g. some top tips/swaps; a sample 7 day menu plan); as well as optimising the following within the programme:<br>Goals and Planning (goal setting: behaviour; action planning)<br>Feedback & Monitoring (self-monitoring of behaviour e.g. complete food diary and get feedback from consultant)<br>Shaping Knowledge (instruction on how to perform a behaviour)<br>Repetition/Substitution (behaviour practice and rehearsal; graded tasks)
**Opportunity**	Social context/accessibility	• Identified with group members/social bonds<br>• Positive relationships with group consultants, personalised support<br>• Supported by partners, childcare/sharing SW meals.	• Didn't bond/socially identify with group<br>• Consultants unsympathetic to postnatal challenges<br>• Partner lack of support/partial adaptation to previous meals.	In the leaflet/brief consultation provided at randomisation, women can be reminded that they can change groups and consultants if desired; incorporate anticipation of impact of the programme on the family/partner into brief consultation (e.g. how to discuss with partner; a section in the leaflet for partner/family to read).<br>Social support (unspecified; practical; emotional)<br>Reward and Threat (social incentive: explore whether their family/peers will congratulate them if they lose weight/attend SW sessions; restructure the social environment e.g. by involving those who will be supportive of them attending SW, of dietary changes and weight loss)

COREQ (COnsolidated criteria for REporting Qualitative research) Checklist

A checklist of items that should be included in reports of qualitative research. You must report the page number in your manuscript where you consider each of the items listed in this checklist. If you have not included this information, either revise your manuscript accordingly before submitting or note N/A.

Topic	Item No.	Guide Questions/Description	Reported on Page No.
**Domain 1: Research team and reflexivity**			
Personal characteristics			
Interviewer/facilitator	1	Which author/s conducted the interview or focus group?	5
Credentials	2	What were the researcher's credentials? E.g. PhD, MD	5
Occupation	3	What was their occupation at the time of the study?	1
Gender	4	Was the researcher male or female?	5
Experience and training	5	What experience or training did the researcher have?	5
Relationship with participants			
Relationship established	6	Was a relationship established prior to study commencement?	5
Participant knowledge of the interviewer	7	What did the participants know about the researcher? e.g. personal goals, reasons for doing the research	5
Interviewer characteristics	8	What characteristics were reported about the inter viewer/facilitator? e.g. Bias, assumptions, reasons and interests in the research topic	5
**Domain 2: Study design**			
Theoretical framework			
Methodological orientation and Theory	9	What methodological orientation was stated to underpin the study? e.g. grounded theory, discourse analysis, ethnography, phenomenology, content analysis	6
Participant selection			
Sampling	10	How were participants selected? e.g. purposive, convenience, consecutive, snowball	5
Method of approach	11	How were participants approached? e.g. face-to-face, telephone, mail, email	5
Sample size	12	How many participants were in the study?	7
Non-participation	13	How many people refused to participate or dropped out? Reasons?	5
Setting			
Setting of data collection	14	Where was the data collected? e.g. home, clinic, workplace	5
Presence of non-participants	15	Was anyone else present besides the participants and researchers?	5
Description of sample	16	What are the important characteristics of the sample? e.g. demographic data, date	17
Data collection			
Interview guide	17	Were questions, prompts, guides provided by the authors? Was it pilot tested?	5
Repeat interviews	18	Were repeat inter views carried out? If yes, how many?	n/a
Audio/visual recording	19	Did the research use audio or visual recording to collect the data?	6
Field notes	20	Were field notes made during and/or after the inter view or focus group?	no
Duration	21	What was the duration of the inter views or focus group?	6
Data saturation	22	Was data saturation discussed?	5
Transcripts returned	23	Were transcripts returned to participants for comment and/or	no

For peer review only

For peer review only - http://bmjopen.bmj.com/site/about/guidelines.xhtml

Topic	Item No.	Guide Questions/Description	Reported on Page No.
		correction?	
**Domain 3: analysis and findings**			
Data analysis			
Number of data coders	24	How many data coders coded the data?	6
Description of the coding tree	25	Did authors provide a description of the coding tree?	6
Derivation of themes	26	Were themes identified in advance or derived from the data?	6
Software	27	What software, if applicable, was used to manage the data?	n/a
Participant checking	28	Did participants provide feedback on the findings?	no
Reporting			
Quotations presented	29	Were participant quotations presented to illustrate the themes/findings? Was each quotation identified? e.g. participant number	7-11
Data and findings consistent	30	Was there consistency between the data presented and the findings?	yes
Clarity of major themes	31	Were major themes clearly presented in the findings?	7
Clarity of minor themes	32	Is there a description of diverse cases or discussion of minor themes?	7

Developed from: Tong A, Sainsbury P, Craig J. Consolidated criteria for reporting qualitative research (COREQ): a 32-item checklist for interviews and focus groups. *International Journal for Quality in Health Care*. 2007. Volume 19, Number 6: pp. 349 – 357

Once you have completed this checklist, please save a copy and upload it as part of your submission. DO NOT include this checklist as part of the main manuscript document. It must be uploaded as a separate file.

For peer review only

BMJ Open

Barriers and facilitators to uptake and retention of inner-city ethnically diverse women in a postnatal weight management intervention: a mixed methods process evaluation within a feasibility trial

Journal:	*BMJ Open*
Manuscript ID	bmjopen-2019-034747.R1
Article Type:	Original research
Date Submitted by the Author:	06-Feb-2020

Complete List of Authors:	Taylor, Cath; University of Surrey, School of Health Sciences<br>Bhavnani, Vanita; National Childbirth Trust<br>Zasada, Magdalena; University of Surrey, School of Health Sciences<br>Ussher, Michael; St George's University of London, Population Health Research Institute; University of Stirling, Institute of Social Marketing and Health<br>Bick, Debra; University of Warwick, Warwick Clinical Trials Unit, Warwick Medical School

**Primary Subject Heading**:	Obstetrics and gynaecology
Secondary Subject Heading:	General practice / Family practice, Public health, Reproductive medicine
Keywords:	Maternal medicine < OBSTETRICS, PUBLIC HEALTH, QUALITATIVE RESEARCH

SCHOLARONE™
Manuscripts

I, the Submitting Author has the right to grant and does grant on behalf of all authors of the Work (as defined in the below author licence), an exclusive licence and/or a non-exclusive licence for contributions from authors who are: i) UK Crown employees; ii) where BMJ has agreed a CC-BY licence shall apply, and/or iii) in accordance with the terms applicable for US Federal Government officers or employees acting as part of their official duties; on a worldwide, perpetual, irrevocable, royalty-free basis to BMJ Publishing Group Ltd ("BMJ") its licensees and where the relevant Journal is co-owned by BMJ to the co-owners of the Journal, to publish the Work in this journal and any other BMJ products and to exploit all rights, as set out in our licence.

The Submitting Author accepts and understands that any supply made under these terms is made by BMJ to the Submitting Author unless you are acting as an employee on behalf of your employer or a postgraduate student of an affiliated institution which is paying any applicable article publishing charge ("APC") for Open Access articles. Where the Submitting Author wishes to make the Work available on an Open Access basis (and intends to pay the relevant APC), the terms of reuse of such Open Access shall be governed by a Creative Commons licence – details of these licences and which Creative Commons licence will apply to this Work are set out in our licence referred to above.

Other than as permitted in any relevant BMJ Author's Self Archiving Policies, I confirm this Work has not been accepted for publication elsewhere, is not being considered for publication elsewhere and does not duplicate material already published. I confirm all authors consent to publication of this Work and authorise the granting of this licence.

For peer review only

Barriers and facilitators to uptake and retention of inner-city ethnically diverse women in a postnatal weight management intervention: a mixed methods process evaluation within a feasibility trial.

Cath Taylor[1]; Vanita Bhavnani[2]; Magdalena Zasada[1]; Michael Ussher[3,4]; Debra Bick[5] and on behalf of the SWAN trial team

[1] School of Health Sciences, University of Surrey, Guildford UK

[2] National Childbirth Trust, London UK

[3] Population Health Research Institute, St George's, University of London, London UK

[4] Institute for Social Marking and Health, University of Stirling, Stirling UK

[5] Warwick Clinical Trials Unit, Warwick Medical School, University of Warwick, Coventry UK

Corresponding author:

Cath Taylor

School of Health Sciences

University of Surrey

Guildford GU2 7YH

Email: cath.taylor@surrey.ac.uk

Tel: +44 1483 688862

Word Count: 3981

Keywords: postpartum; maternal health; weight management; health behaviour

1

For peer review only

Abstract

Objectives: To understand the barriers and facilitators to uptake and retention of postnatal women randomised to a commercial group weight management intervention using the COM-B (Capability, Opportunity, Motivation - Behaviour) behaviour change model.

Design: Concurrent mixed methods (qualitative dominant) process evaluation nested within a feasibility randomised controlled trial, comprising questionnaires and interviews at six and 12 months post-birth.

Setting: One National Health Service maternity unit in an inner city area in the south of England.

Participants: 98 postnatal women with BMIs>25kg/m^2 (overweight/obese) at pregnancy commencement.

Intervention: Twelve week Slimming World® (SW) commercial group weight management programme, commencing anytime from 8 to 16 weeks postnatally.

Primary and secondary outcome measures: Data regarding uptake and retention from questionnaires and interviews conducted six and 12 months post birth, analysed thematically and mapped to the COM-B model.

Results: Barriers to SW uptake mostly concerned opportunity issues (e.g., lack of time or childcare support) though some women also lacked motivation, not feeling that weight reduction was a priority, and a few cited capability issues such as lacking confidence. Weight loss aspirations were also a key factor explaining retention, as were social opportunity issues, particularly in relation to factors such as the extent of group identity and relationship with the group consultant; and physical opportunity such as perceived support from and fit with family lifestyle. In addition, barriers relating to beliefs and expectations about the SW programme were identified, including concerns regarding compatibility with breastfeeding and importance of exercise. Women's understanding of the SW approach, and capability to implement into their lifestyles, appeared related to level of attendance (dose-response effect).

Conclusions: Uptake and retention in commercial weight management programmes may be enhanced by applying behaviour change techniques to address the barriers impacting on women's perceived capability, motivation and opportunity to participate.

Trial registration: This is the process evaluation of a feasibility RCT (ISRCTN 39186148)

For peer review only - http://bmjopen.bmj.com/site/about/guidelines.xhtml

Article summary

Strengths and limitations of this study

- The need to consider postnatal weight management interventions has been highlighted by the lack of benefit shown by pregnancy interventions. This is this first study to use behaviour change theory to explore uptake and retention of postnatal women in a weight management intervention
- The sample were from an ethnically and economically diverse inner-city population
- The intervention is an evidence-based commercially available programme available throughout the UK and in several other countries worldwide
- As a single centre feasibility study, findings cannot necessarily be generalised
- Techniques proposed to mitigate the emergent barriers require further evaluation.

Introduction

The proportion of women entering pregnancy with an overweight or obese Body Mass Index (i.e. BMIs of ≥25kg/m^2) is high and increasing, accounting for approximately half of all pregnancies in the UK, [1] and USA.[2] Pregnant women with obese BMIs have an increased risk of adverse outcomes for themselves (e.g. pre-eclampsia, gestational diabetes) and their infants (e.g. stillbirth, congenital anomalies).[3] Furthermore, retaining weight postnatally is associated with other poor health behaviours including smoking, poor diet, lack of exercise and not breastfeeding.[4-6] It also increases risks for long-term obesity, hypertension, diabetes and degenerative joint disease in women,[7]. The infants of women with obese BMIs in pregnancy are also at risk of higher BMI and blood pressure themselves in childhood and young adulthood.[6]

Intervening prior to pregnancy is challenging as most pregnancies are unplanned[8] and women are not generally in touch with health services until they are pregnant. Due to limited evidence of effective weight management interventions during pregnancy[9] attention has shifted to the postnatal period. However, studies of postnatal weight management have methodological limitations (e.g. lacking information on randomisation or response rates), and wide heterogeneity in relation to intervention and evaluation.[10-11] Furthermore, neither uptake nor retention are explicitly reported in these reviews.

In general populations, commercial weight management programmes were more effective than health service delivered programmes,[12] with the advantage of being nationally available and tailored to community needs and settings. Few cater for women in pregnancy or postpartum; one

For peer review only - http://bmjopen.bmj.com/site/about/guidelines.xhtml

exception is Slimming World (SW, www.slimmingworld.co.uk) who have produced guidelines and support in collaboration with the Royal College of Midwifery.

According to the COM-B (capability, opportunity, motivation and behaviour) model,[13] successful behaviour change, such as weight management, requires an individual to be *capable* of performing the behaviour, have the *opportunity* to carry out the behaviour, and be *motivated* to change the behaviour. For general populations, COM-B has been applied in a review of uptake and retention in weight management programmes (including SW).[14] Uptake was greatest in those motivated to attend and this was highly related to capability (knowledge and skills to change behaviour) and opportunity, particularly social opportunity. Social opportunity (including within the programme; e.g. social support, supportive leader, and outside; e.g. family and friends) was the dominant driver of retention, with psychological capability also important (highest retention when there was self-monitoring and feedback on behaviour). A recently completed feasibility randomised controlled trial of access to SW in a postnatal population[15] presented data on recruitment, retention and other feasibility outcomes, which were supportive of progression to a definitive trial. This paper reports results from the mixed methods process evaluation of the study, the objective being to use the COM-B to examine the barriers and facilitators to uptake and retention of women allocated to the intervention.

Methods

Reported according to COREQ guidelines.

Design: Concurrent mixed methods (qualitative dominant) process evaluation nested within a feasibility randomised controlled trial, comprising questionnaires and interviews at six and 12 months post-birth. The study was conducted between 01/12/2015 – 30/11/2018.

Setting: One NHS maternity unit in an ethnically diverse inner city area in the south of England with high levels of socioeconomic disadvantage, mobility and migration.

Participants: Postnatal women aged ≥18 with BMIs≥25kg/m^2 at pregnancy commencement or with normal BMIs (18.5kg/m^2-24.9kg/m^2) who met criteria for excessive gestational weight gain (EGWG) at 36 weeks gestation, randomly allocated at 36 weeks gestation to access 12-weekly SW sessions, commenced anytime from 8-16 weeks post-birth (n=98). Full inclusion/exclusion criteria are published elsewhere[15].

Intervention: Slimming World® (Alfreton, UK), is a commercial weight management programme. The programme is underpinned by behaviour change theory and techniques, including social cognitive

For peer review only - http://bmjopen.bmj.com/site/about/guidelines.xhtml

theory[16] with a focus on motivation and self-efficacy for weight management and reducing relapse. Key techniques include goal setting, self-monitoring, social support, and positive reinforcement.[17-18] Consultants leading the groups receive standardised training from SW dieticians and nutritionists, which includes motivational skills to support positive lifestyle changes, nutrition, food facts, and role of exercise and activity in health and weight management. Groups follow a standard format, starting with members being weighed (confidentially), whilst new members attend an introductory session, followed by group session (called Image Therapy) where members discuss their experiences of weight management that week, facilitated by the consultant and aimed at supporting behaviour change, and encouraging peer support. Sessions can include basic cooking skills, taking cost, cultural preferences and time constraints into account. Physical activity is encouraged and rewarded through 'body magic awards', including redefining what 'activity' can include (e.g. housework, gardening). Members also have access to SW online resources (weight tracking, recipes, tips, motivational articles etc). The dietary plan encourages most foods (80%) to be fruit, vegetables, carbohydrates and lean protein; a smaller but 'mandatory' allowance for calcium-rich and fibre-rich foods; and a daily optional allowance for foods high in fat or sugar. Women who are breastfeeding have an increased allowance for calcium-rich foods.

Women allocated to the intervention were offered (fees waived) the standard SW membership (12 sessions over 14 weeks). SW guidance is that in order to achieve a 5% weight loss, a difference considered to improve health outcomes, individuals need to attend at least 10 of 12 sessions.[19] In our process evaluation, we considered whether women stayed for whole group sessions (or left after being weighed), and whether women attended 10+ sessions, 6-9 sessions; 1-5 sessions or did not attend at all. On completion of the 12 weeks, women could continue with standard SW fees applicable. Women received an information leaflet about joining SW, a congratulations text from the Research Midwives on the birth of their baby, and reminder texts at 6-8 weeks postnatally. From 8-16 weeks postnatally they could call SW to register and locate their nearest groups.

Questionnaire component: All women were invited to complete questionnaires at baseline (36 weeks gestation) and six months post-birth. Responders at six-months were invited to complete a further questionnaire at 12 months post-birth. The six and 12 month questionnaires, which included measures of dietary intake, mental and physical health and lifestyle behaviours (reported elsewhere, 15), also assessed attendance at SW. This included uptake (asking if they attended any sessions and, if so, age of their baby when attending their first session); and retention (how many sessions attended in total; if they stayed for the whole session or just got weighed). Two open questions asked why they did not stay for whole sessions (if relevant), and how useful they found the sessions. Women that had not attended any sessions were asked to explain why they chose not to attend.

For peer review only - http://bmjopen.bmj.com/site/about/guidelines.xhtml

Interview component: Interviews were conducted by a female experienced qualitative researcher (VB) with a purposively selected sample of women at approximately six months post-birth and 12 months post-birth. Criteria for inviting women captured diversity (in line with guidance[20]) in relation to: attendance at SW sessions (women who attended 10+ sessions vs. attended fewer or did not attend at all); weight change; and ethnicity). The same criteria were applied at 12 months post-birth, but also included six women from the initial sample to provide a nested longitudinal sample. The topic guide included: motivation for study participation, experiences of the intervention, and reasons for uptake and retention (or not), with prompts informed by the COM-B model. Women were approached initially by Research Midwives who provided a study information sheet. With consent, their contact details were shared with the researcher who contacted them to answer questions, confirm consent and arrange interviews. All interviews were by telephone (face-to-face was offered), and lasted between 20-50 minutes (average 45 minutes). Interviews were-audio recorded. Barriers and facilitators to study participation were considered separately to those that relate to the intervention and are reported elsewhere [15].

Analysis:

Quantitative questionnaire data were analysed descriptively. Analysis of open questions and interview data was underpinned by the COM-B model[13] and used the Framework method[21]. Interviews were transcribed verbatim. Transcripts were read by two researchers (CT & MZ for questionnaires; CT & VB for interviews), who independently noted key themes. Coding was compared and discussed before final coding and labelling. Data were examined to identify themes in relation to capability, motivation and opportunity factors (e.g. comparing women that attended different 'doses' of sessions; and those that lost or gained weight). Data were examined longitudinally if women completed both questionnaires/interviews. As the COM-B components are not mutually exclusive, data often fit several dimensions.

Integration of findings: Data from questionnaires and interviews were analysed separately before being compared, and integrated into a single framework. Key barriers to uptake and retention were mapped to behaviour change techniques for enhancing uptake and retention.

Quotations are anonymised with pseudonyms (flowers). Each quote is labelled with the pseudonym, number of sessions attended, and weight change from baseline to the time point they provided data.

For peer review only - http://bmjopen.bmj.com/site/about/guidelines.xhtml

Patient and Public Involvement:

The choice of SW as the intervention was heavily influenced by feedback from local women who had participated in a trial of weight management during pregnancy.[22] Their views included that the intervention should be non-NHS, peer-supported to enable social support but not just 'new mums' (though knowledgeable of their needs), flexible, and suitable for taking their babies. Four women who had recently given birth and experienced weight management issues were recruited to join our PPI group via advertisements in community midwifery locations. They were supported by a researcher (VB) to contribute to the development of study materials, conduct of the trial, interpretation and dissemination of findings.

Ethics approval was granted by the Health Research Authority London – Camberwell St Giles REC (16/LO/1422).

Results

Of 98 women randomised to the intervention, 83 (85%) completed the six month questionnaire, 69 of whom (69/83, 83%) also completed the 12 month questionnaire. At least one of the two questions about SW was answered by all women who attended at least one SW session. Fifty-two (53%) women did not attend any sessions and 39 (75%) gave a reason for not attending.

Interviews were conducted with 13 women immediately post-intervention (approximately six to eight months post-birth); and with nine women (six of whom had participated at six-months) at 12 months post-birth. The characteristics of participants are provided in Table 1.

Uptake

Just over half of the intervention women did not attend any SW sessions. Most commonly this related to 'opportunity', particularly lack of time to attend. Some had difficulties organising childcare or fitting SW sessions into their family schedule, for example sessions clashing with older children's bedtimes. One woman reported breastfeeding on demand as a limiting factor, either not knowing she could take her baby with her (or not choosing to do so):

> *"I chose to breastfeed on demand so I would not have been able to attend any scheduled sessions easily."* [Rose; gained 12kg 12mth]

For peer review only - http://bmjopen.bmj.com/site/about/guidelines.xhtml

Two participants found the offer too soon post-birth; stating that six months postpartum would have been easier. Other 'opportunity' factors included their own or their infants ill health, or social factors including lack of confidence (capability) due to perceived language barriers reported by a non-native English speaker, and others stating they were leaving the country for an extended period, or leaving the city and not realising that they could attend groups elsewhere. Two participants experienced errors in registration that prevented them from enrolling in time. The location of the groups was only mentioned as a barrier by five women.

In addition to these 'opportunity' factors, there were 'motivational' barriers to uptake for some women who were either happy with their weight, stated they had already lost weight, or did not feel motivated to lose weight. Others did not believe SW to be suitable, either because they did not believe it promoted an effective or balanced diet, or due to a perceived lack of relevance for new mums:

> *"I was asked whether I would be comfortable with people cheering if I'd lost weight which made me think I had done something wrong rather than have a child! And also that the programme was targeted to teach me how to eat better- e.g. by using olive oil and not eating ready meals - which I'd never done. So it sounded more like something for unhealthy people with bad habits, not new mums."* [Lily; lost 6.5kg 12mth]

Non-attendance was more prevalent amongst non-White British/Irish women. The reasons given for this were predominantly 'opportunity' issues regarding accessibility such as childcare/lack of fit with routine. Similar reasons were given by White British/Irish women who did not attend any sessions. Only two women that did not attend any sessions commented on aspects of the diet presenting barriers (one White European background, and the other Asian).

Retention

Although nearly half of women assigned to SW attended at least one session (46/98, 47%), only half of these (n=19/46) attended the full 'dose' (i.e. 10+ sessions); the mean number of sessions was 6.74 (SD 3.94). Virtually all women who only attended a few sessions attended these in consecutive weeks. Those who attended greater numbers of sessions (e.g. 5-9 sessions) sometimes had one or two week gaps. Five key themes emerged which differed across the three groups (10+, 6-9 and 1-5 sessions) and help to explain barriers and facilitators to retention. Each is considered below and summarized in Table 2 where they are mapped to behaviour change techniques (BCTs) that may mitigate barriers.

For peer review only - http://bmjopen.bmj.com/site/about/guidelines.xhtml

For peer review only

Motivation: weight loss aspirations

Women attending 10+ sessions, and some women attending fewer sessions but losing weight over the data collection period, tended to perceive themselves as overweight, to understand why they gained weight, and to perceive weight loss as a priority.

> *"I guess I got into the routine of comfort eating and putting on more weight. I thought that if I didn't take this opportunity I would have really been at a loss."* [Violet; 11; lost 17.5kg 12mth]

In contrast, women attending 6-9 sessions and gaining weight, although acknowledging they were overweight and understanding why, were less likely to prioritise weight loss; they commonly said that adapting to life with their baby was more important:

> *"For me three months into having had a baby, I wasn't standing there thinking I've got to lose the weight. I was standing there thinking, wow how do I reacclimatise and recalibrate my life to accommodate this little person."* [Aster; 6; gained 2.8kg 12mth]

Weight reduction was also given low priority by women attending 1-5 sessions, who were less likely to recognise they were overweight, and/or state they did not gain much weight during their pregnancy, as the rationale for not prioritising weight reduction, compared with women attending more sessions.

Motivation (psychological capability): Beliefs and expectations regarding weight loss

A common theme among women attending 10+ sessions was that the intervention had altered their beliefs about how to lose weight, particularly regarding the role of exercise:

> "*I've gone on various diets but half-heartedly, and I've always done it though exercise … So it's a kind of revelation that it's 90% food and 10% exercise which is what I learnt at Slimming World … seeing 5½ lbs loss in the first week confirmed it would work. I am proud of myself.*" [Heather; 12; lost 2.6kg 12mth]

Women who attended fewer SW sessions were more likely to question the intervention, often because they felt it failed to prioritise exercise as they had expected.

> "*I just felt there should also be an activity, there wasn't any exercise classes or anything like that and maybe that side of things didn't attract me*" [Iris; 1; gained 4.4kg 12mth]

Other beliefs that explained poor retention included that SW was unsuitable for breastfeeding mothers, or that their needs were broader than weight management (e.g. also requiring postnatal mental-wellbeing support).

For peer review only - http://bmjopen.bmj.com/site/about/guidelines.xhtml

Motivation/Capability: Understanding and implementing the intervention

A key 'capability' factor was the extent to which women understood and were able to implement the SW programme. Women who attended 10+ sessions described following the dietary plan and using SW online resources to support them. They were motivated by the health and wellbeing benefits they experienced for themselves and for their families:

> *"It felt to me to be sustainable, not a harsh diet that I was only going to do for a few weeks. I was still enjoying myself and enjoying my food and I was still losing weight, so that was very motivating."* [Hibiscus; 10; gained 0.3kg 12mth]

In contrast, women who attended 6-9 sessions appeared to have a poorer understanding of the SW plan and/or reported difficulties adapting to the plan:

> *"It's quite a low sugar, low fat diet and I was reluctant to cut my fat levels too much so I tried to keep some full fat milk and I would still eat more fatty avocados. I just took the free food and went with it without actually counting syns…I am not convinced that sticking to the plan would actually help me maintain longer term."* [Lavender; 9; lost 0.4kg 12mth]

Many gained weight and their motivation to attend may have been affected by lack of perceived benefit. For women who attended 1-5 sessions these barriers were most apparent, suggesting that there might be a 'dose-response' effect: the more sessions women attended, the more likely it was that they would feel 'capable' and thereby 'motivated' to successfully implement lifestyle changes.

Social opportunity and Motivation: Social context

Opportunity factors relating to both the internal (intervention) and external (home support) social context were important explanatory factors for retention. Internally, women who attended 10+ sessions were most likely to describe identifying with other SW group members, seeing their group consultants as helpful in adapting the programme to their circumstances (e.g. breastfeeding) and generally perceived the groups as a supportive and safe environment:

> *"I was positively surprised how much the group was supportive and I'm also new in the area so it was a really good way to meet people, like meet new mums. I keep bumping into members in the supermarket and in the park exercising …. I felt part of a little community"* [Orchid; 10; lost 3.7kg 12mth]

> "*Consultant very positive, made baby welcome and was supportive*" [Peony; lost 3.1kg 12mth]

For peer review only - http://bmjopen.bmj.com/site/about/guidelines.xhtml

Those who attended <10 sessions were more likely to express negative views about their groups or consultants. Several mentioned having difficulty bonding or socially identifying with other group members, or that their consultants were unsympathetic to their specific postnatal challenges:

> "*The atmosphere was very city/work based and not welcoming to new mothers*" [Poinsettia; 1; gained 1.8kg 6mth]

> *"The woman who ran the group, wasn't very nice, she was very critical…"* [Hyacinth; 7; gained 13.7kg 12mth]

In relation to 'external' factors, women attending 10+ sessions commonly reported benefitting from support from their partners, either practically (e.g. childcare whilst they attended), or willingness to adapt their diets alongside their partner. One husband also joined SW: *"I took my husband with me. He lost quite a lot of weight as well. So it was good."* [Orchid; 10; lost 3.7kg 12mth]

For others, the lack of partners' support presented a barrier to participating and/or losing weight:

> *"It's a lot easier to do* [follow the plan] *on the nights he* [husband] *is working. I suppose he just likes certain food … he likes to have a proper meal."* [Hibiscus; 10; gained 0.3kg 12mth]

> "Husband not supportive" [Poinsettia; 1; gained 1.8kg 6mth]

Physical opportunity: Accessibility of the intervention

Most women (35/46, 76%) stayed at the same group for all their sessions, the majority attending groups on weekdays (40/46, 87%) though the time of day was split fairly equally between morning and evening attendance. A key opportunity factor was the accessibility of the intervention, which either facilitated or created a barrier to attendance. Issues included *timing of onset* of the intervention, *ease of attendance* and *duration of the intervention*. Women had diverse views that were unrelated to the number of sessions attended or their weight change. In relation to intervention onset, most reported that starting SW between 8-16 weeks post-birth was acceptable. However some felt it was too early in the postnatal period, coinciding with the time they might be experiencing difficulties with breastfeeding, concerns over their baby's health and the general transition to parenthood:

> "*couldn't concentrate while had baby with me as breastfeeding … offer too early in postnatal period, bad timing of groups with baby, exhausted*" [Primrose; 2; lost 0.7kg 12mth]

For peer review only - http://bmjopen.bmj.com/site/about/guidelines.xhtml

Regarding ease of attendance, most women could access a SW group that was located and timed to suit them. However, two women who attended <10 sessions, stated having difficulties in this regard:

> *"That* [group] *was the only one within walking distance from my house...On most days; I didn't stay [*after being weighed*]...because of the inconvenient timing she was by that point in time she would be really ratty and ready for sleep."* [Hyacinth; 7; gained 13.7kg 12mth]

Other 'opportunity' reasons for missing sessions and/or not completing the programme included holiday plans, illness (in woman or infant), and childcare difficulties (for siblings or where they didn't want to take their babies with them).

The third key accessibility issue concerned the intervention duration. Many who completed the programme considered it to be too short:

> "*I don't think 12 weeks is long enough to see any real benefits and to establish healthy eating/lose weight long term. I would say 6 months is optimal*" [Tulip; 6; gained 3.6kg 12mth].

Women could continue beyond 12 weeks but fees were applicable. Some women did this, for others the timing of the intervention ceasing presented a major barrier as coincided with the end of maternity benefit:

> *"Once the 12 weeks were up … that was the time when I [had] no money, so £5 a week ended up being quite a lot of money that I couldn't really afford … if it was possible to do it for a year [during] maternity leave that would be fantastic."* Azalea; 11; lost -1.8kg 12mth]

None of the women who responded to questionnaire/s or participated in interview(s) mentioned cost as a facilitator to uptake or retention. White British/Irish women were more likely to attend 10+ sessions than women from other ethnic groups. Examination of the barriers between these groups indicated that women from non-White British/Irish groups were more likely to mention accessibility opportunity factors such as time, access, and childcare as barriers to retention, as opposed to the White British/Irish women who were more likely to mention social context opportunity factors such as the consultant or group identity. We found no clear relationship between socio-economic variables (household income or IMD decile) and uptake or retention, though all negative comments regarding the diet (n=5) were from women in the highest income groups.

For peer review only - http://bmjopen.bmj.com/site/about/guidelines.xhtml

Discussion

This is the first study to use behaviour change theory to explore uptake and retention in a postnatal weight management intervention. A number of modifiable barriers to uptake and retention were identified. Fewer than half of the intervention arm women attended at least one session; a rate consistent with weight management studies in pregnancy[23-24] and general population.[25] Reported barriers could potentially be addressed using BCTs (see Table 2) - for example through strengthening motivation to lose weight by tackling incompatible beliefs and/or supporting consideration of the pros and cons of weight loss and providing evidence regarding health consequences. Previous studies of weight management in pregnancy have highlighted the difficulty that midwives and other health professionals have discussing weight with women,[26-28] and indicate a training need to support effective conversations. Some women also found the timing of the intervention challenging: offering a wider commencement window (e.g. allowing women to start SW anytime up to six months post-birth) may have increased uptake.

Only 19% of intervention arm women received the 'full dose' of 10+ sessions. Retention (and uptake) may be enhanced by offering enhanced support to help women adapt the dietary plan to their lifestyle and family needs, to address capability issues (e.g. understanding and/or implementing the diet); and opportunity factors might be mitigated by ensuring women know there is flexibility regarding the group they attend; and by encouraging women to identify those in their social environment who will support their dietary changes and weight loss aspirations. This enhanced support may need to be tailored for different ethnic groups. There appeared to be a 'dose-response' relationship with women attending 10+ sessions reporting the greatest benefit. This concurs with findings from a general population study where those attending at least 75% of the offered sessions (also provided by SW) achieved the greatest weight loss.[19] Women who did not receive the full dose often had erroneous perceptions about the programme and its effectiveness or compatibility with their postnatal status, which in turn reinforced their decision to give up. The use of techniques to shape knowledge regarding effective weight loss and the SW programme at the time of study recruitment may mitigate this barrier and more closely align their expectations with the programme. As well as widening the commencement window, findings suggest that a longer duration of intervention may improve the sustainability of weight loss, particularly as the current duration ended at a similar time to women's statutory maternity leave payments ending. Recent evidence from an RCT comparing weight loss in adults with obese BMIs allocated to brief (booklet only), 12 week or 52 week programmes found the 52 week programme was both more clinically and cost effective than shorter programmes.[29]

To our knowledge this is the first study that has used behaviour change theory to understand uptake and retention of postnatal women in a weight management programme, and included a diverse

For peer review only - http://bmjopen.bmj.com/site/about/guidelines.xhtml

For peer review only

inner-city population with women with various levels of attendance. The COM-B was originally developed to inform intervention development, though has been applied to evaluation of interventions in various settings [30-33]. Its use in this feasibility study enables both evaluation and design, and findings will inform changes to enhance uptake and retention for the intervention in the main study. The use of framework analysis ensured data analysis was transparent and enabled all research team members to contribute. Whilst the interview sample was small, the integration of the survey and interview findings is a strength of this study. Whilst only a small number of women who did not take up the intervention were interviewed, 78% (42/51) of those who did not attend completed a survey where they explained why. The interviews were via telephone rather than face-to-face and this is a potential limitation. However, all the women said that they preferred telephone to face-to-face. Moreover, we consider that telephone interviews were appropriate as the topic of the interviews were not covering sensitive topics that might especially warrant face-to-face, nor was it seen as necessary to gather contextual information about the participant's environment. It is not surprising that women preferred telephone interviews as this is a practical option when caring for a young infant. The findings regarding the potential impact of ethnicity on uptake and retention require further exploration in a larger study. As a single centre feasibility study, findings cannot be generalised, though the intervention is commercially available throughout the UK and in several countries worldwide, which may enhance the relevance of findings. The analysis and interpretation of uptake and retention is limited to that which might optimise the intervention for a future trial. Discussions with women with BMI ≥25 kg/m^2 who had recently used maternity services in the local area informed the choice of intervention: they had a clear preference for non-NHS setting and groups that were not solely for women with babies. However, evidence from this study suggests that some women may have preferred NHS settings and groups focussing on women with infants. Findings will be used to optimise the potency of the intervention in a future trial, with an embedded process evaluation to further evaluate the impact of proposed BCTs on uptake and retention.

Author contributions

CT and DB developed the study concept and study design, with the support of MU. VB completed data collection and VB, CT and MZ were responsible for data analysis. All authors contributed to interpretation of findings. DB had primary responsibility for overseeing the overall SWAN trial. CT drafted the first version of the manuscript, with the support of MU, MZ and DB. All authors read and approved the final manuscript.

For peer review only - http://bmjopen.bmj.com/site/about/guidelines.xhtml

SWAN trial team members:

Dr Amanda Avery, University of Nottingham

Dr Andy Healey, King's College London

Ms Nina Khazaededah, Guy's and St Thomas' NHS Foundation Trust

Dr Sara McMullen, National Childbirth Trust

Professor Eugene Oteng-Ntim, King's College London

Dr Bimpe Oki, Lambeth and Southwark Public Health

Professor Lucilla Poston, King's College London

Mr Paul Seed, King's College London

Acknowledgements:

We thank the women who participated in this study, and Sheila O'Connor and Victoria Craig (research midwives) for supporting recruitment and data collection. We would also like to thank members of our trial steering group.

Funding:

This study is funded by the National Institute for Health Research (NIHR) Public Health Research Programme (ref no. 14/67/14). The views expressed are those of the author(s) and not necessarily those of the NIHR or the Department of Health and Social Care.

Competing interests:

AA (a member of the SWAN trial team), alongside her academic position at the University of Nottingham, also holds a consultancy position at Slimming World®(Alfreton, UK). Neither AA nor Slimming World® (Alfreton, UK) had access to the study data or were involved in data collection or analyses. DB was supported by the National Institute for Health Research (NIHR) Collaboration for Leadership in Applied Health Research and Care South London (NIHR CLAHRC South London) at King's College Hospital NHS Foundation Trust. The other authors declare that they have no competing interests in relation to this study.

Data Sharing Statement:

The data sets generated and/or analysed as part of this study are not publicly available due to them containing information that could compromise research participant privacy/consent but are available from the corresponding author on reasonable request.

References

1. NHS Maternity Statistics 2017-18 https://files.digital.nhs.uk/C3/47466E/hosp-epis-stat-mat-summary-report%202017-18.pdf Last accessed: October 2019
2. Dudenhausen JW 2015 Prepregnancy body weight and gestational weight gain. *Am J Obstet Gynecol* 2015;591-92
3. Leddy MA, Power ML, Schulkin J The Impact of Maternal Obesity on Maternal and Fetal Health. *Rev Obstet Gynecol* 2008;1(4):170-178

For peer review only - http://bmjopen.bmj.com/site/about/guidelines.xhtml

4. Hilson JA, Rasmussen KM, Kjolhede CL. Excessive weight gain during pregnancy is associated with earlier termination of breast-feeding among white women. *The Journal of Nutrition* 2006;136(1):140–146.
5. Vinter CA, Jensen DM, Ovesen P, et al. Postpartum weight retention and breastfeeding among obese women from the randomized controlled Lifestyle in Pregnancy (LiP) trial. *Acta Obstet Gynecol Scand* 2014;93(8):794-801
6. Poston L, Harthoorn LF, van der Beek EM: Obesity in pregnancy: implications for the mother and lifelong health of the child. A consensus statement. *Pediatr Res* 2011;69:175–180.
7. Rooney BL, Schauberger CW, Mathiason MA. Impact of perinatal weight change on long-term obesity and obesity-related illnesses. *Obstet Gynecol* 2005;106:1349–56.
8. Hanson M, Godfrey K, Poston L, et al Pre-conception Health. In: Davies SC Annual Report of the Chief Medical Officer, 2014, The health of the 51%: women. London: Department of Health (2015)
9. Poston L. Obesity in pregnancy: could lifestyle interventions work? *BMC Medicine* 2014;12:201
10. Amorim Adegboye AR, Linne YM. Diet or exercise, or both, for weight reduction in women after childbirth. *Cochrane Database of Systematic Reviews* 2013, Issue 7. Art. No.: CD005627. DOI: 10.1002/14651858.CD005627.pub3.
11. van der Pligt P, Willcox J, Hesketh KD, et al. Systematic review of lifestyle interventions to limit postpartum weight retention: implications for future opportunities to prevent maternal overweight and obesity following childbirth. *Obesity Reviews* 2013;14:792–805 doi: 10.1111/obr.12053
12. Jolly K, Lewis A, Beach J, et al Comparison of range of commercial or primary care led weight reduction programmes with minimal intervention control for weight loss in obesity: Lighten Up randomised controlled trial. *BMJ* 2011;343:d6500 doi: 10.1136/bmj.d6500
13. Michie S, van Strelen M, West R. The behaviour change wheel: A new method for characterising and designing behaviour change interventions. *Implementation Science* 2011;6:42. doi: 10 1186/1748-5908-6-42.
14. Gidlow C, Povey R, Ellis N, et al Uptake and retention in group-based weight management services. Literature review and behavioural analysis. Final report 2018. Public Health England: London. 2018.
15. Bick D, Taylor C, Bhavnani V, Healey A, Seed P, Roberts S et al (2020) Lifestyle information and commercial weight management groups to support maternal postnatal weight management and positive lifestyle behaviour: the SWAN feasibility randomised controlled trial. BJOG DOI: 10.1111/1471-0528.16043
16. Bandura, A *Social foundations of thought and action: A social cognitive theory*. Prentice-Hall, Inc. 1986.
17. Elfhag K, Rössner S: Who succeeds in maintaining weight loss? A conceptual review of factors associated with weight loss maintenance and weight regain. *Obes Rev* 2005;6(1):67–85.
18. Wing RR, Jeffery RW: Benefits of recruiting participants with friends and increasing social support for weight loss and maintenance. *J Consult Clin Psychol* 1999;67(1):132–138
19. Stubbs RJ, Morris L, Pallister C, et al Weight outcomes audit in 1.3 million adults during their first 3 months' attendance in a commercial weight management programme *BMC Public Health* 2015;15:882
20. O'Cathain A, Hoddinott P, Lewin S, et al. Maximising the impact of qualitative research in feasibility studies for randomised controlled trials: guidance for researchers *Pilot and Feasibility Studies* 2015;1:32

For peer review only - http://bmjopen.bmj.com/site/about/guidelines.xhtml

21. Ritchie J, Lewis J: Qualitative research practice: a guide for social science students and researchers. London: Sage 2003
22. Poston L, Bell R, Croker H, et al on behalf of the UPBEAT Trial Consortium. Effect of a behavioural intervention in obese pregnant women (the UPBEAT study): a multicentre, randomised controlled trial. *Lancet Diabetes Endocrinol* 2015;3(10):767–777
23. Davis, DL, Raymond, JE, Clements, V, et al Addressing obesity in pregnancy: The design and feasibility of an innovative intervention in NSW, Australia. *Women Birth* 2012;25:174– 80.
24. Knight, BA, Wyatt, K. Barriers encountered when recruiting obese pregnant women to a dietary intervention. *Nursing Times*. 2010;106:20– 2
25. Blane DN, McLoone P,Morrison D, *et al*. Patient and practice characteristics predicting attendance and completion at a specialist weight management service in the UK: a cross sectional study. *BMJ Open* 2017;7:e018286. doi:10.1136/bmjopen-2017-018286
26. Holton S, East C, Fisher J. Weight management during pregnancy: a qualitative study of women's and care providers' experiences and perspectives *BMC Pregnancy and Childbirth* 2017;17:351
27. Olander EK, Atkinson L, Edmunds JK, et al The views of pre- and post-natal women and health professionals regarding gestational weight gain: An exploratory study *Sexual & Reproductive Healthcare* 2011;2:43–48
28. Flannery C, McHugh S, Kenny LC, *et al*. Exploring obstetricians', midwives' and general practitioners' approach to weight management in pregnant women with a BMI ≥25 kg/m2: a qualitative study. *BMJ Open* 2019;9:e024808. doi:10.1136/bmjopen-2018-024808
29. Ahern AL, Wheeler GM, Aveyard P, et al Extended and standard duration weight-loss programme referrals for adults in primary care (WRAP): a randomised controlled trial *Lancet* 2017;389:2214–25
30. Craig LE, McInnes E, Taylor N, Grimley R, Cadilhac DA, Considine J, Middleton S. Identifying the barriers and enablers for a triage, treatment, and transfer clinical intervention to manage acute stroke patients in the emergency department: a systematic review using the theoretical domains framework (TDF). Implement Sci. 2016;11:157.
31. Graham-Rowe E, Lorencatto F, Lawrenson J, Burr J, Grimshaw J, Ivers N, Presseau J, Vale L, Peto T, Bunce C. Barriers to and enablers of diabetic retinopathy screening attendance: a systematic review of published and grey literature. Diabet Med. 2018;35:1308-19.
32. Heslehurst N, Newham J, Maniatopoulos G, Fleetwood C, Robalino S, Rankin J. Implementation of pregnancy weight management and obesity guidelines: a meta-synthesis of healthcare professionals' barriers and facilitators using the Theoretical D omains F ramework. Obes Rev. 2014;15:462-86.
33. McDonagh LK, Saunders JM, Cassell J, Curtis T, Bastaki H, Hartney T, Rait G (2018) Application of the COM-B model to barriers and facilitators to chlamydia testing in general practice for young people and primary care practitioners: a systematic review. Implementation Science, 13:130. https://doi.org/10.1186/s13012-018-0821-y

For peer review only - http://bmjopen.bmj.com/site/about/guidelines.xhtml

Table 1: Characteristics of study participants

Characteristics	Intervention group n=98	Survey response n=83*	Interview 6 months n=13	Interview 12 months n=9
**Ethnicity**				
White	49 (50%)	43 (52%)	10 (77%)	6 (67%)
Black	35 (36%)	27 (33%)	3 (23%)	3 (33%)
Asian	8 (8%)	8 (10%)	0 (0%)	0 (0%)
Other	6 (6%)	3 (4%)	0 (0%)	0 (0%)
Unknown	0 (0%)	2 (2%)	0 (0%)	0 (0%)
**Age**				
20-24	7 (7%)	4 (5%)	1 (8%)	0 (0%)
25-29	19 (19%)	17 (20%)	3 (23%)	1 (11%)
30-34	33 (34%)	26 (31%)	4 (31%)	3 (33%)
35-39	33 (34%)	30 (36%)	4 (31%)	4 (44%)
40-44	6 (6%)	6 (7%)	1 (8%)	1 (11%)
**BMI at booking**				
<25 with EGWG**	3 (3%)	3 (4%)	0 (0%)	1 (11%)
25-29.9	55 (56%)	51 (61%)	9 (69%)	6 (67%)
>=30	40 (41%)	29 (35%)	4 (31%)	2 (22%)
**Parity**				
Primigravida	50 (51%)	43 (52%)	9 (69%)	5 (56%)
Multigravida	46(47%)	38 (46%)	3 (23%)	4 (44%)
Unknown	2 (2%)	2 (2%)	1 (8%)	0 (0%)
**Number of sessions attended (data from SW)**				
10+	19 (19%)	19 (23%)	6 (46%)	4 (44%)
6 to 9	8 (8%)	8 (10%)	3 (23%)	1 (11%)
1 to 5	19 (19%)	15 (18%)	3 (23%)	3 (33%)
None	52 (53%)	41 (49%)	1 (8%)	1 (11%)

*includes those who responded either to six month questionnaire only, or to both six and 12 month questionnaires

** EGWG Excessive Gestational Weight Gain

For peer review only

Table 2: Barriers to uptake and retention mapped to COM-B and to potential behaviour change techniques

COM-B	Theme	10+ Slimming World sessions	<10 Slimming World sessions	Behaviour change techniques that could help mitigate barriers
**Motivation**	weight loss aspirations	• self-perceived as overweight<br>• understood why gained weight<br>• desired to lose weight after birth	• some acknowledged overweight and understood why but viewed weight management as less of a priority<br>• Others did not self-perceive as overweight	Provide information (leaflet/brief consultation) at outset to help influence knowledge and understanding regarding weight loss and consequences of action/no action.<br>• Identity (Framing/reframing; Incompatible beliefs; identity associated with changed behaviour)<br>• Comparison of outcomes (Pros and cons; credible source; comparative imagining of future outcomes)<br>• Goal setting (problem solving)<br>• Natural consequences (Information about health consequences; Salience of consequences; Information about social and environmental consequences
**Capability/ Motivation**	beliefs and expectations about SW	• SW and resulting weight loss helped change any pre-existing ideas about how to lose weight, esp re: role of exercise	• Mistrustful of SW, in part as perceived as failing to prioritise exercise in line with pre-existing beliefs, seen as unsuitable for breastfeeding women, or felt gap in care e.g in relation to postnatal mental wellbeing	Leaflet/brief consultation (as above) to include:<br>• Shaping knowledge (Information about antecedents e.g. provide reliable info regarding weight gain/loss; Re-attribution e.g. elicit perceived causes of weight gain/how to lose weight and suggest alternatives)<br>• Goals and Planning (goal setting: behaviour e.g. to make clear at the outset that the SW programme considers the role of exercise but focusses on diet; that 10+ sessions are needed for it to be most effective; Goal setting: outcome – make clear at the outset that the outcome goals are focussed on weight management, but that weight loss may have other positive outcomes such as improved sense of wellbeing/mental health.

For peer review only - http://bmjopen.bmj.com/site/about/guidelines.xhtml

				• Comparison of Outcomes (Pros and cons: discuss pros and cons of exercise vs diet for weight loss)
**Capability**	understanding and implementing SW	• Understood dietary plan, found easy to follow, planned meals and used online resources.<br>• Considered plan unrestrictive, sustainable and compatible with postnatal lifestyle<br>• Positive benefits for them and their families	• Did not appear to understand the dietary plan, only partial implementation, and experienced difficulties making dietary adjustments.	Development of a bespoke booklet to give to women at randomisation that summarizes key elements of the programme (e.g. some top tips/swaps; a sample 7 day menu plan); as well as optimising the following within the programme:<br>Goals and Planning (goal setting: behaviour; action planning)<br>Feedback & Monitoring (self-monitoring of behaviour e.g. complete food diary and get feedback from consultant)<br>Shaping Knowledge (instruction on how to perform a behaviour)<br>Repetition/Substitution (behaviour practice and rehearsal; graded tasks)
**Opportunity**	Social context/accessibility	• Identified with group members/social bonds<br>• Positive relationships with group consultants, personalised support<br>• Supported by partners, childcare/sharing SW meals.	• Didn't bond/socially identify with group<br>• Consultants unsympathetic to postnatal challenges<br>• Partner lack of support/partial adaptation to previous meals.	In the leaflet/brief consultation provided at randomisation, women can be reminded that they can change groups and consultants if desired; incorporate anticipation of impact of the programme on the family/partner into brief consultation (e.g. how to discuss with partner; a section in the leaflet for partner/family to read).<br>Social support (unspecified; practical; emotional)<br>Reward and Threat (social incentive: explore whether their family/peers will congratulate them if they lose weight/attend SW sessions; restructure the social environment e.g. by involving those who will be supportive of them attending SW, of dietary changes and weight loss)

COREQ (COnsolidated criteria for REporting Qualitative research) Checklist

A checklist of items that should be included in reports of qualitative research. You must report the page number in your manuscript where you consider each of the items listed in this checklist. If you have not included this information, either revise your manuscript accordingly before submitting or note N/A.

Topic	Item No.	Guide Questions/Description	Reported on Page No.
**Domain 1: Research team and reflexivity**			
Personal characteristics			
Interviewer/facilitator	1	Which author/s conducted the interview or focus group?	5
Credentials	2	What were the researcher's credentials? E.g. PhD, MD	5
Occupation	3	What was their occupation at the time of the study?	1
Gender	4	Was the researcher male or female?	5
Experience and training	5	What experience or training did the researcher have?	5
Relationship with participants			
Relationship established	6	Was a relationship established prior to study commencement?	5
Participant knowledge of the interviewer	7	What did the participants know about the researcher? e.g. personal goals, reasons for doing the research	5
Interviewer characteristics	8	What characteristics were reported about the inter viewer/facilitator? e.g. Bias, assumptions, reasons and interests in the research topic	5
**Domain 2: Study design**			
Theoretical framework			
Methodological orientation and Theory	9	What methodological orientation was stated to underpin the study? e.g. grounded theory, discourse analysis, ethnography, phenomenology, content analysis	6
Participant selection			
Sampling	10	How were participants selected? e.g. purposive, convenience, consecutive, snowball	5
Method of approach	11	How were participants approached? e.g. face-to-face, telephone, mail, email	5
Sample size	12	How many participants were in the study?	7
Non-participation	13	How many people refused to participate or dropped out? Reasons?	5
Setting			
Setting of data collection	14	Where was the data collected? e.g. home, clinic, workplace	5
Presence of non-participants	15	Was anyone else present besides the participants and researchers?	5
Description of sample	16	What are the important characteristics of the sample? e.g. demographic data, date	17
Data collection			
Interview guide	17	Were questions, prompts, guides provided by the authors? Was it pilot tested?	5
Repeat interviews	18	Were repeat inter views carried out? If yes, how many?	n/a
Audio/visual recording	19	Did the research use audio or visual recording to collect the data?	6
Field notes	20	Were field notes made during and/or after the inter view or focus group?	no
Duration	21	What was the duration of the inter views or focus group?	6
Data saturation	22	Was data saturation discussed?	5
Transcripts returned	23	Were transcripts returned to participants for comment and/or	no

For peer review only

For peer review only - http://bmjopen.bmj.com/site/about/guidelines.xhtml

Topic	Item No.	Guide Questions/Description	Reported on Page No.
		correction?	
**Domain 3: analysis and findings**			
Data analysis			
Number of data coders	24	How many data coders coded the data?	6
Description of the coding tree	25	Did authors provide a description of the coding tree?	6
Derivation of themes	26	Were themes identified in advance or derived from the data?	6
Software	27	What software, if applicable, was used to manage the data?	n/a
Participant checking	28	Did participants provide feedback on the findings?	no
Reporting			
Quotations presented	29	Were participant quotations presented to illustrate the themes/findings? Was each quotation identified? e.g. participant number	7-11
Data and findings consistent	30	Was there consistency between the data presented and the findings?	yes
Clarity of major themes	31	Were major themes clearly presented in the findings?	7
Clarity of minor themes	32	Is there a description of diverse cases or discussion of minor themes?	7

Developed from: Tong A, Sainsbury P, Craig J. Consolidated criteria for reporting qualitative research (COREQ): a 32-item checklist for interviews and focus groups. *International Journal for Quality in Health Care*. 2007. Volume 19, Number 6: pp. 349 – 357

Once you have completed this checklist, please save a copy and upload it as part of your submission. DO NOT include this checklist as part of the main manuscript document. It must be uploaded as a separate file.

For peer review only

BMJ Open

Barriers and facilitators to uptake and retention of inner-city ethnically diverse women in a postnatal weight management intervention: a mixed methods process evaluation within a feasibility trial in England

Journal:	*BMJ Open*
Manuscript ID	bmjopen-2019-034747.R2
Article Type:	Original research
Date Submitted by the Author:	22-May-2020
Complete List of Authors:	Taylor, Cath; University of Surrey, School of Health Sciences<br>Bhavnani, Vanita; National Childbirth Trust<br>Zasada, Magdalena; University of Surrey, School of Health Sciences<br>Ussher, Michael; St George's University of London, Population Health Research Institute; University of Stirling, Institute of Social Marketing and Health<br>Bick, Debra; University of Warwick, Warwick Clinical Trials Unit, Warwick Medical School
**Primary Subject Heading**:	Obstetrics and gynaecology
Secondary Subject Heading:	General practice / Family practice, Public health, Reproductive medicine
Keywords:	Maternal medicine < OBSTETRICS, PUBLIC HEALTH, QUALITATIVE RESEARCH

SCHOLARONE™
Manuscripts

I, the Submitting Author has the right to grant and does grant on behalf of all authors of the Work (as defined in the below author licence), an exclusive licence and/or a non-exclusive licence for contributions from authors who are: i) UK Crown employees; ii) where BMJ has agreed a CC-BY licence shall apply, and/or iii) in accordance with the terms applicable for US Federal Government officers or employees acting as part of their official duties; on a worldwide, perpetual, irrevocable, royalty-free basis to BMJ Publishing Group Ltd ("BMJ") its licensees and where the relevant Journal is co-owned by BMJ to the co-owners of the Journal, to publish the Work in this journal and any other BMJ products and to exploit all rights, as set out in our licence.

The Submitting Author accepts and understands that any supply made under these terms is made by BMJ to the Submitting Author unless you are acting as an employee on behalf of your employer or a postgraduate student of an affiliated institution which is paying any applicable article publishing charge ("APC") for Open Access articles. Where the Submitting Author wishes to make the Work available on an Open Access basis (and intends to pay the relevant APC), the terms of reuse of such Open Access shall be governed by a Creative Commons licence – details of these licences and which Creative Commons licence will apply to this Work are set out in our licence referred to above.

Other than as permitted in any relevant BMJ Author's Self Archiving Policies, I confirm this Work has not been accepted for publication elsewhere, is not being considered for publication elsewhere and does not duplicate material already published. I confirm all authors consent to publication of this Work and authorise the granting of this licence.

For peer review only

Barriers and facilitators to uptake and retention of inner-city ethnically diverse women in a postnatal weight management intervention: a mixed methods process evaluation within a feasibility trial in England.

Cath Taylor[1]; Vanita Bhavnani[2]; Magdalena Zasada[1]; Michael Ussher[3,4]; Debra Bick[5] and on behalf of the SWAN trial team

[1] School of Health Sciences, University of Surrey, Guildford UK

[2] National Childbirth Trust, London UK

[3] Population Health Research Institute, St George's, University of London, London UK

[4] Institute for Social Marketing and Health, University of Stirling, Stirling UK

[5] Warwick Clinical Trials Unit, Warwick Medical School, University of Warwick, Coventry UK

Corresponding author:

Cath Taylor

School of Health Sciences

University of Surrey

Guildford GU2 7YH

Email: cath.taylor@surrey.ac.uk

Tel: +44 1483 688862

Word Count: 3981

Keywords: postpartum; maternal health; weight management; health behaviour

For peer review only - http://bmjopen.bmj.com/site/about/guidelines.xhtml

Abstract

Objectives: To understand the barriers and facilitators to uptake and retention of postnatal women randomised to a commercial group weight management intervention using the COM-B (Capability, Opportunity, Motivation - Behaviour) behaviour change model.

Design: Concurrent mixed methods (qualitative dominant) process evaluation nested within a feasibility randomised controlled trial, comprising questionnaires and interviews at six and 12 months post-birth.

Setting: One National Health Service maternity unit in an inner city area in the south of England.

Participants: 98 postnatal women with BMIs>25kg/m^2 (overweight/obese) at pregnancy commencement.

Intervention: Twelve week Slimming World® (SW) commercial group weight management programme, commencing anytime from 8 to 16 weeks postnatally.

Primary and secondary outcome measures: Data regarding uptake and retention from questionnaires and interviews conducted six and 12 months post birth, analysed thematically and mapped to the COM-B model.

Results: Barriers to SW uptake mostly concerned opportunity issues (e.g., lack of time or childcare support) though some women also lacked motivation, not feeling that weight reduction was a priority, and a few cited capability issues such as lacking confidence. Weight loss aspirations were also a key factor explaining retention, as were social opportunity issues, particularly in relation to factors such as the extent of group identity and relationship with the group consultant; and physical opportunity such as perceived support from and fit with family lifestyle. In addition, barriers relating to beliefs and expectations about the SW programme were identified, including concerns regarding compatibility with breastfeeding and importance of exercise. Women's understanding of the SW approach, and capability to implement into their lifestyles, appeared related to level of attendance (dose-response effect).

Conclusions: Uptake and retention in commercial weight management programmes may be enhanced by applying behaviour change techniques to address the barriers impacting on women's perceived capability, motivation and opportunity to participate.

Trial registration: This is the process evaluation of a feasibility RCT (ISRCTN 39186148)

For peer review only - http://bmjopen.bmj.com/site/about/guidelines.xhtml

Article summary

Strengths and limitations of this study

- This is this first study to use behaviour change theory to explore uptake and retention of postnatal women in a weight management intervention
- The sample were from an ethnically and economically diverse inner-city population
- The intervention is an evidence-based commercially available programme available throughout the UK and in several other countries worldwide
- As a single centre feasibility study, findings cannot necessarily be generalised
- Techniques proposed to mitigate the emergent barriers require further evaluation.

Introduction

The proportion of women entering pregnancy with an overweight or obese Body Mass Index (i.e. BMIs of ≥25kg/m^2) is high and increasing, accounting for approximately half of all pregnancies in the UK, [1] and USA.[2] Pregnant women with obese BMIs have an increased risk of adverse outcomes for themselves (e.g. pre-eclampsia, gestational diabetes) and their infants (e.g. stillbirth, congenital anomalies).[3] Furthermore, retaining weight postnatally is associated with other poor health behaviours including smoking, poor diet, lack of exercise and not breastfeeding.[4-6] It also increases risks for long-term obesity, hypertension, diabetes and degenerative joint disease in women,[7]. The infants of women with obese BMIs in pregnancy are also at risk of higher BMI and blood pressure themselves in childhood and young adulthood.[6]

Intervening prior to pregnancy is challenging as most pregnancies are unplanned[8] and women are not generally in touch with health services until they are pregnant. Due to limited evidence of effective weight management interventions during pregnancy[9] attention has shifted to the postnatal period. However, studies of postnatal weight management have methodological limitations (e.g. lacking information on randomisation or response rates), and wide heterogeneity in relation to intervention and evaluation.[10-11] Furthermore, neither uptake nor retention are explicitly reported in these reviews.

In general populations, commercial weight management programmes were more effective than health service delivered programmes,[12] with the advantage of being nationally available and tailored to community needs and settings. Few cater for women in pregnancy or postpartum; one

For peer review only - http://bmjopen.bmj.com/site/about/guidelines.xhtml

exception is Slimming World (SW, www.slimmingworld.co.uk) who have produced guidelines and support in collaboration with the Royal College of Midwifery.

According to the COM-B (capability, opportunity, motivation and behaviour) model,[13] successful behaviour change, such as weight management, requires an individual to be *capable* of performing the behaviour, have the *opportunity* to carry out the behaviour, and be *motivated* to change the behaviour. For general populations, COM-B has been applied in a review of uptake and retention in weight management programmes (including SW).[14] Uptake was greatest in those motivated to attend and this was highly related to capability (knowledge and skills to change behaviour) and opportunity, particularly social opportunity. Social opportunity (including within the programme; e.g. social support, supportive leader, and outside; e.g. family and friends) was the dominant driver of retention, with psychological capability also important (highest retention when there was self-monitoring and feedback on behaviour). A recently completed feasibility randomised controlled trial of access to SW in a postnatal population[15] presented data on recruitment, retention and other feasibility outcomes, which were supportive of progression to a definitive trial. This paper reports results from the mixed methods process evaluation of the study, the objective being to use the COM-B to examine the barriers and facilitators to uptake and retention of women allocated to the intervention.

Methods

Reported according to COREQ guidelines.

Design: Concurrent mixed methods (qualitative dominant) process evaluation nested within a feasibility randomised controlled trial, comprising questionnaires and interviews at six and 12 months post-birth. The study was conducted between 01/12/2015 – 30/11/2018.

Setting: One NHS maternity unit in an ethnically diverse inner city area in the south of England with high levels of socioeconomic disadvantage, mobility and migration.

Participants: Postnatal women aged ≥18 with BMIs≥25kg/m^2 at pregnancy commencement or with normal BMIs (18.5kg/m^2-24.9kg/m^2) who met criteria for excessive gestational weight gain (EGWG) at 36 weeks gestation, randomly allocated at 36 weeks gestation to access 12-weekly SW sessions, commenced anytime from 8-16 weeks post-birth (n=98). Full inclusion/exclusion criteria are published elsewhere[15].

For peer review only - http://bmjopen.bmj.com/site/about/guidelines.xhtml

Intervention: Slimming World® (Alfreton, UK), is a commercial weight management programme. The programme is underpinned by behaviour change theory and techniques, including social cognitive theory[16] with a focus on motivation and self-efficacy for weight management and reducing relapse. Key techniques include goal setting, self-monitoring, social support, and positive reinforcement.[17-18] Consultants leading the groups receive standardised training from SW dieticians and nutritionists, which includes motivational skills to support positive lifestyle changes, nutrition, food facts, and role of exercise and activity in health and weight management. Groups follow a standard format, starting with members being weighed (confidentially), whilst new members attend an introductory session, followed by group session (called Image Therapy) where members discuss their experiences of weight management that week, facilitated by the consultant and aimed at supporting behaviour change, and encouraging peer support. Sessions can include basic cooking skills, taking cost, cultural preferences and time constraints into account. Physical activity is encouraged and rewarded through 'body magic awards', including redefining what 'activity' can include (e.g. housework, gardening). Members also have access to SW online resources (weight tracking, recipes, tips, motivational articles etc). The dietary plan encourages most foods (80%) to be fruit, vegetables, carbohydrates and lean protein; a smaller but 'mandatory' allowance for calcium-rich and fibre-rich foods; and a daily optional allowance for foods high in fat or sugar. Women who are breastfeeding have an increased allowance for calcium-rich foods.

Women allocated to the intervention were offered (fees waived) the standard SW membership (12 sessions over 14 weeks). SW guidance is that in order to achieve a 5% weight loss, a difference considered to improve health outcomes, individuals need to attend at least 10 of 12 sessions.[19] In our process evaluation, we considered whether women stayed for whole group sessions (or left after being weighed), and whether women attended 10+ sessions, 6-9 sessions; 1-5 sessions or did not attend at all. On completion of the 12 weeks, women could continue with standard SW fees applicable. Women received an information leaflet about joining SW, a congratulations text from the Research Midwives on the birth of their baby, and reminder texts at 6-8 weeks postnatally. From 8-16 weeks postnatally they could call SW to register and locate their nearest groups.

Questionnaire component: All women were invited to complete questionnaires at baseline (36 weeks gestation) and six months post-birth. Responders at six-months were invited to complete a further questionnaire at 12 months post-birth. The six and 12 month questionnaires, which included measures of dietary intake, mental and physical health and lifestyle behaviours (reported elsewhere, 15), also assessed attendance at SW. This included uptake (asking if they attended any sessions and, if so, age of their baby when attending their first session); and retention (how many sessions attended in total; if they stayed for the whole session or just got weighed). Two open questions

For peer review only - http://bmjopen.bmj.com/site/about/guidelines.xhtml

asked why they did not stay for whole sessions (if relevant), and how useful they found the sessions. Women that had not attended any sessions were asked to explain why they chose not to attend.

Interview component: Interviews were conducted by a female experienced qualitative researcher (VB) with a purposively selected sample of women at approximately six months post-birth and 12 months post-birth. Criteria for inviting women captured diversity (in line with guidance[20]) in relation to: attendance at SW sessions (women who attended 10+ sessions vs. attended fewer or did not attend at all); weight change; and ethnicity). The same criteria were applied at 12 months post-birth, but also included six women from the initial sample to provide a nested longitudinal sample. The topic guide included: motivation for study participation, experiences of the intervention, and reasons for uptake and retention (or not), with prompts informed by the COM-B model. All interviews were by telephone (face-to-face was offered), and lasted between 20-50 minutes (average 45 minutes). Interviews were-audio recorded. Barriers and facilitators to study participation were considered separately to those that relate to the intervention and are reported elsewhere [15].

Analysis:

Quantitative questionnaire data were analysed descriptively. Analysis of open questions and interview data was underpinned by the COM-B model[13] and used the Framework method[21]. Interviews were transcribed verbatim. Transcripts were read by two researchers (CT & MZ for questionnaires; CT & VB for interviews), who independently noted key themes. Coding was compared and discussed before final coding and labelling. Data were examined to identify themes in relation to capability, motivation and opportunity factors (e.g. comparing women that attended different ‘doses’ of sessions; and those that lost or gained weight). Data were examined longitudinally if women completed both questionnaires/interviews. As the COM-B components are not mutually exclusive, data often fit several dimensions.

Integration of findings: Data from questionnaires and interviews were analysed separately before being compared, and integrated into a single framework. Key barriers to uptake and retention were mapped to behaviour change techniques for enhancing uptake and retention.

Quotations are anonymised with pseudonyms (flowers). Each quote is labelled with the pseudonym, number of sessions attended, and weight change from baseline to the time point they provided data.

For peer review only - http://bmjopen.bmj.com/site/about/guidelines.xhtml

Patient and Public Involvement:

The choice of SW as the intervention was heavily influenced by feedback from local women who had participated in a trial of weight management during pregnancy.[22] Their views included that the intervention should be non-NHS, peer-supported to enable social support but not just 'new mums' (though knowledgeable of their needs), flexible, and suitable for taking their babies. Four women who had recently given birth and experienced weight management issues were recruited to join our PPI group via advertisements in community midwifery locations. They were supported by a researcher (VB) to contribute to the development of study materials, conduct of the trial, interpretation and dissemination of findings.

Ethical Approval

Ethics approval was granted by the Health Research Authority London – Camberwell St Giles REC (16/LO/1422). Women who met the purposive criteria were approached initially by Research Midwives who provided a study information sheet and consent form (either in face-to-face contacts or by post). Consent forms were either returned in person to the Research Midwives or posted directly to the researcher. Those who consented were then contacted by the researcher to answer questions, re-confirm consent verbally and arrange interviews. Consent was also reconfirmed verbally at the start of interviews.

Results

Of 98 women randomised to the intervention, 83 (85%) completed the six month questionnaire, 69 of whom (69/83, 83%) also completed the 12 month questionnaire. At least one of the two questions about SW was answered by all women who attended at least one SW session. Fifty-two (53%) women did not attend any sessions and 39 (75%) gave a reason for not attending.

Interviews were conducted with 13 women immediately post-intervention (approximately six to eight months post-birth); and with nine women (six of whom had participated at six-months) at 12 months post-birth. The characteristics of participants are provided in Table 1.

Uptake

Just over half of the intervention women did not attend any SW sessions. Most commonly this related to 'opportunity', particularly lack of time to attend. Some had difficulties organising childcare

For peer review only - http://bmjopen.bmj.com/site/about/guidelines.xhtml

or fitting SW sessions into their family schedule, for example sessions clashing with older children's bedtimes. One woman reported breastfeeding on demand as a limiting factor, either not knowing she could take her baby with her (or not choosing to do so):

> *"I chose to breastfeed on demand so I would not have been able to attend any scheduled sessions easily."* [Rose; gained 12kg 12mth]

Two participants found the offer too soon post-birth; stating that six months postpartum would have been easier. Other 'opportunity' factors included their own or their infants ill health, or social factors including lack of confidence (capability) due to perceived language barriers reported by a non-native English speaker, and others stating they were leaving the country for an extended period, or leaving the city and not realising that they could attend groups elsewhere. Two participants experienced errors in registration that prevented them from enrolling in time. The location of the groups was only mentioned as a barrier by five women.

In addition to these 'opportunity' factors, there were 'motivational' barriers to uptake for some women who were either happy with their weight, stated they had already lost weight, or did not feel motivated to lose weight. Others did not believe SW to be suitable, either because they did not believe it promoted an effective or balanced diet, or due to a perceived lack of relevance for new mums:

> *"I was asked whether I would be comfortable with people cheering if I'd lost weight which made me think I had done something wrong rather than have a child! And also that the programme was targeted to teach me how to eat better- e.g. by using olive oil and not eating ready meals - which I'd never done. So it sounded more like something for unhealthy people with bad habits, not new mums."* [Lily; lost 6.5kg 12mth]

For peer review only - http://bmjopen.bmj.com/site/about/guidelines.xhtml

Non-attendance was more prevalent amongst non-White British/Irish women. The reasons given for this were predominantly ‘opportunity’ issues regarding accessibility such as childcare/lack of fit with routine. Similar reasons were given by White British/Irish women who did not attend any sessions. Only two women that did not attend any sessions commented on aspects of the diet presenting barriers (one White European background, and the other Asian).

Retention

Although nearly half of women assigned to SW attended at least one session (46/98, 47%), only half of these (n=19/46) attended the full ‘dose’ (i.e. 10+ sessions); the mean number of sessions was 6.74 (SD 3.94). Virtually all women who only attended a few sessions attended these in consecutive weeks. Those who attended greater numbers of sessions (e.g. 5-9 sessions) sometimes had one or two week gaps. Five key themes emerged which differed across the three groups (10+, 6-9 and 1-5 sessions) and help to explain barriers and facilitators to retention. Each is considered below and summarized in Table 2 where they are mapped to behaviour change techniques (BCTs) that may mitigate barriers.

Motivation: weight loss aspirations

Women attending 10+ sessions, and some women attending fewer sessions but losing weight over the data collection period, tended to perceive themselves as overweight, to understand why they gained weight, and to perceive weight loss as a priority.

> *“I guess I got into the routine of comfort eating and putting on more weight. I thought that if I didn’t take this opportunity I would have really been at a loss.”* [Violet; 11; lost 17.5kg 12mth]

In contrast, women attending 6-9 sessions and gaining weight, although acknowledging they were overweight and understanding why, were less likely to prioritise weight loss; they commonly said that adapting to life with their baby was more important:

> *“For me three months into having had a baby, I wasn’t standing there thinking I’ve got to lose the weight. I was standing there thinking, wow how do I reacclimatise and recalibrate my life to accommodate this little person.”* [Aster; 6; gained 2.8kg 12mth]

Weight reduction was also given low priority by women attending 1-5 sessions, who were less likely to recognise they were overweight, and/or state they did not gain much weight during their

For peer review only - http://bmjopen.bmj.com/site/about/guidelines.xhtml

pregnancy, as the rationale for not prioritising weight reduction, compared with women attending more sessions.

Motivation (psychological capability): Beliefs and expectations regarding weight loss

A common theme among women attending 10+ sessions was that the intervention had altered their beliefs about how to lose weight, particularly regarding the role of exercise:

> *"I've gone on various diets but half-heartedly, and I've always done it though exercise … So it's a kind of revelation that it's 90% food and 10% exercise which is what I learnt at Slimming World … seeing 5½ lbs loss in the first week confirmed it would work. I am proud of myself."* [Heather; 12; lost 2.6kg 12mth]

Women who attended fewer SW sessions were more likely to question the intervention, often because they felt it failed to prioritise exercise as they had expected.

> *"I just felt there should also be an activity, there wasn't any exercise classes or anything like that and maybe that side of things didn't attract me"* [Iris; 1; gained 4.4kg 12mth]

Other beliefs that explained poor retention included that SW was unsuitable for breastfeeding mothers, or that their needs were broader than weight management (e.g. also requiring postnatal mental-wellbeing support).

Motivation/Capability: Understanding and implementing the intervention

A key 'capability' factor was the extent to which women understood and were able to implement the SW programme. Women who attended 10+ sessions described following the dietary plan and using SW online resources to support them. They were motivated by the health and wellbeing benefits they experienced for themselves and for their families:

> *"It felt to me to be sustainable, not a harsh diet that I was only going to do for a few weeks. I was still enjoying myself and enjoying my food and I was still losing weight, so that was very motivating."* [Hibiscus; 10; gained 0.3kg 12mth]

In contrast, women who attended 6-9 sessions appeared to have a poorer understanding of the SW plan and/or reported difficulties adapting to the plan:

> *"It's quite a low sugar, low fat diet and I was reluctant to cut my fat levels too much so I tried to keep some full fat milk and I would still eat more fatty avocados. I just took the free food and went with it without actually counting syns…I am not convinced that sticking to the plan would actually help me maintain longer term."* [Lavender; 9; lost 0.4kg 12mth]

For peer review only - http://bmjopen.bmj.com/site/about/guidelines.xhtml

Many gained weight and their motivation to attend may have been affected by lack of perceived benefit. For women who attended 1-5 sessions these barriers were most apparent, suggesting that there might be a 'dose-response' effect: the more sessions women attended, the more likely it was that they would feel 'capable' and thereby 'motivated' to successfully implement lifestyle changes.

Social opportunity and Motivation: Social context

Opportunity factors relating to both the internal (intervention) and external (home support) social context were important explanatory factors for retention. Internally, women who attended 10+ sessions were most likely to describe identifying with other SW group members, seeing their group consultants as helpful in adapting the programme to their circumstances (e.g. breastfeeding) and generally perceived the groups as a supportive and safe environment:

> *"I was positively surprised how much the group was supportive and I'm also new in the area so it was a really good way to meet people, like meet new mums. I keep bumping into members in the supermarket and in the park exercising …. I felt part of a little community"* [Orchid; 10; lost 3.7kg 12mth]

> "*Consultant very positive, made baby welcome and was supportive*" [Peony; lost 3.1kg 12mth]

Those who attended <10 sessions were more likely to express negative views about their groups or consultants. Several mentioned having difficulty bonding or socially identifying with other group members, or that their consultants were unsympathetic to their specific postnatal challenges:

> "*The atmosphere was very city/work based and not welcoming to new mothers*" [Poinsettia; 1; gained 1.8kg 6mth]

> *"The woman who ran the group, wasn't very nice, she was very critical…"* [Hyacinth; 7; gained 13.7kg 12mth]

In relation to 'external' factors, women attending 10+ sessions commonly reported benefitting from support from their partners, either practically (e.g. childcare whilst they attended), or willingness to adapt their diets alongside their partner. One husband also joined SW: *"I took my husband with me. He lost quite a lot of weight as well. So it was good."* [Orchid; 10; lost 3.7kg 12mth]

For others, the lack of partners' support presented a barrier to participating and/or losing weight:

For peer review only - http://bmjopen.bmj.com/site/about/guidelines.xhtml

"It's a lot easier to do [follow the plan] *on the nights he* [husband] *is working. I suppose he just likes certain food … he likes to have a proper meal."* [Hibiscus; 10; gained 0.3kg 12mth]

"Husband not supportive" [Poinsettia; 1; gained 1.8kg 6mth]

Physical opportunity: Accessibility of the intervention

Most women (35/46, 76%) stayed at the same group for all their sessions, the majority attending groups on weekdays (40/46, 87%) though the time of day was split fairly equally between morning and evening attendance. A key opportunity factor was the accessibility of the intervention, which either facilitated or created a barrier to attendance. Issues included *timing of onset* of the intervention, *ease of attendance* and *duration of the intervention*. Women had diverse views that were unrelated to the number of sessions attended or their weight change. In relation to intervention onset, most reported that starting SW between 8-16 weeks post-birth was acceptable. However some felt it was too early in the postnatal period, coinciding with the time they might be experiencing difficulties with breastfeeding, concerns over their baby's health and the general transition to parenthood:

"couldn't concentrate while had baby with me as breastfeeding … offer too early in postnatal period, bad timing of groups with baby, exhausted" [Primrose; 2; lost 0.7kg 12mth]

Regarding ease of attendance, most women could access a SW group that was located and timed to suit them. However, two women who attended <10 sessions, stated having difficulties in this regard:

"That [group] *was the only one within walking distance from my house…On most days; I didn't stay [*after being weighed*]...because of the inconvenient timing she was by that point in time she would be really ratty and ready for sleep."* [Hyacinth; 7; gained 13.7kg 12mth]

Other 'opportunity' reasons for missing sessions and/or not completing the programme included holiday plans, illness (in woman or infant), and childcare difficulties (for siblings or where they didn't want to take their babies with them).

The third key accessibility issue concerned the intervention duration. Many who completed the programme considered it to be too short:

For peer review only - http://bmjopen.bmj.com/site/about/guidelines.xhtml

> *"I don't think 12 weeks is long enough to see any real benefits and to establish healthy eating/lose weight long term. I would say 6 months is optimal"* [Tulip; 6; gained 3.6kg 12mth].

Women could continue beyond 12 weeks but fees were applicable. Some women did this, for others the timing of the intervention ceasing presented a major barrier as coincided with the end of maternity benefit:

> *"Once the 12 weeks were up … that was the time when I [had] no money, so £5 a week ended up being quite a lot of money that I couldn't really afford … if it was possible to do it for a year [during] maternity leave that would be fantastic."* Azalea; 11; lost -1.8kg 12mth]

None of the women who responded to questionnaire/s or participated in interview(s) mentioned cost as a facilitator to uptake or retention. White British/Irish women were more likely to attend 10+ sessions than women from other ethnic groups. Examination of the barriers between these groups indicated that women from non-White British/Irish groups were more likely to mention accessibility opportunity factors such as time, access, and childcare as barriers to retention, as opposed to the White British/Irish women who were more likely to mention social context opportunity factors such as the consultant or group identity. We found no clear relationship between socio-economic variables (household income or IMD decile) and uptake or retention, though all negative comments regarding the diet (n=5) were from women in the highest income groups.

Discussion

This is the first study to use behaviour change theory to explore uptake and retention in a postnatal weight management intervention. A number of modifiable barriers to uptake and retention were identified. Fewer than half of the intervention arm women attended at least one session; a rate consistent with weight management studies in pregnancy[23-24] and general population.[25] Reported barriers could potentially be addressed using BCTs (see Table 2) - for example through strengthening motivation to lose weight by tackling incompatible beliefs and/or supporting consideration of the pros and cons of weight loss and providing evidence regarding health consequences. Previous studies of weight management in pregnancy have highlighted the difficulty that midwives and other health professionals have discussing weight with women,[26-28] and indicate a training need to support effective conversations. Some women also found the timing of the intervention challenging: offering a wider commencement window (e.g. allowing women to start SW anytime up to six months post-birth) may have increased uptake.

For peer review only

Only 19% of intervention arm women received the 'full dose' of 10+ sessions. Retention (and uptake) may be enhanced by offering enhanced support to help women adapt the dietary plan to their lifestyle and family needs, to address capability issues (e.g. understanding and/or implementing the diet); and opportunity factors might be mitigated by ensuring women know there is flexibility regarding the group they attend; and by encouraging women to identify those in their social environment who will support their dietary changes and weight loss aspirations. This enhanced support may need to be tailored for different ethnic groups. There appeared to be a 'dose-response' relationship with women attending 10+ sessions reporting the greatest benefit. This concurs with findings from a general population study where those attending at least 75% of the offered sessions (also provided by SW) achieved the greatest weight loss.[19] Women who did not receive the full dose often had erroneous perceptions about the programme and its effectiveness or compatibility with their postnatal status, which in turn reinforced their decision to give up. The use of techniques to shape knowledge regarding effective weight loss and the SW programme at the time of study recruitment may mitigate this barrier and more closely align their expectations with the programme. As well as widening the commencement window, findings suggest that a longer duration of intervention may improve the sustainability of weight loss, particularly as the current duration ended at a similar time to women's statutory maternity leave payments ending. Recent evidence from an RCT comparing weight loss in adults with obese BMIs allocated to brief (booklet only), 12 week or 52 week programmes found the 52 week programme was both more clinically and cost effective than shorter programmes.[29]

To our knowledge this is the first study that has used behaviour change theory to understand uptake and retention of postnatal women in a weight management programme, and included a diverse inner-city population with women with various levels of attendance. The COM-B was originally developed to inform intervention development, though has been applied to evaluation of interventions in various settings [30-33]. Its use in this feasibility study enables both evaluation and design, and findings will inform changes to enhance uptake and retention for the intervention in the main study. The use of framework analysis ensured data analysis was transparent and enabled all research team members to contribute. Whilst the interview sample was small, the integration of the survey and interview findings is a strength of this study. Whilst only a small number of women who did not take up the intervention were interviewed, 78% (42/51) of those who did not attend completed a survey where they explained why. The interviews were via telephone rather than face-to-face and this is a potential limitation. However, all the women said that they preferred telephone to face-to-face. Moreover, we consider that telephone interviews were appropriate as the topic of the interviews were not covering sensitive topics that might especially warrant face-to-face, nor was

For peer review only - http://bmjopen.bmj.com/site/about/guidelines.xhtml

it seen as necessary to gather contextual information about the participant's environment. It is not surprising that women preferred telephone interviews as this is a practical option when caring for a young infant. The findings regarding the potential impact of ethnicity on uptake and retention require further exploration in a larger study. As a single centre feasibility study, findings cannot be generalised, though the intervention is commercially available throughout the UK and in several countries worldwide, which may enhance the relevance of findings. The analysis and interpretation of uptake and retention is limited to that which might optimise the intervention for a future trial. Discussions with women with BMI ≥25 kg/m^2 who had recently used maternity services in the local area informed the choice of intervention: they had a clear preference for non-NHS setting and groups that were not solely for women with babies. However, evidence from this study suggests that some women may have preferred NHS settings and groups focussing on women with infants. Findings will be used to optimise the potency of the intervention in a future trial, with an embedded process evaluation to further evaluate the impact of proposed BCTs on uptake and retention.

Author contributions

CT and DB developed the study concept and study design, with the support of MU. VB completed data collection and VB, CT and MZ were responsible for data analysis. All authors contributed to interpretation of findings. DB had primary responsibility for overseeing the overall SWAN trial. CT drafted the first version of the manuscript, with the support of MU, MZ and DB. All authors read and approved the final manuscript.

SWAN trial team members:

Dr Amanda Avery, University of Nottingham

Dr Andy Healey, King's College London

Ms Nina Khazaededah, Guy's and St Thomas' NHS Foundation Trust

Dr Sara McMullen, National Childbirth Trust

Professor Eugene Oteng-Ntim, King's College London

Dr Bimpe Oki, Lambeth and Southwark Public Health

Professor Lucilla Poston, King's College London

Mr Paul Seed, King's College London

For peer review only - http://bmjopen.bmj.com/site/about/guidelines.xhtml

Acknowledgements:

We thank the women who participated in this study, and Sheila O'Connor and Victoria Craig (research midwives) for supporting recruitment and data collection. We would also like to thank members of our trial steering group.

Funding:

This study is funded by the National Institute for Health Research (NIHR) Public Health Research Programme (ref no. 14/67/14). The views expressed are those of the author(s) and not necessarily those of the NIHR or the Department of Health and Social Care.

Competing interests:

AA (a member of the SWAN trial team), alongside her academic position at the University of Nottingham, also holds a consultancy position at Slimming World®(Alfreton, UK). Neither AA nor Slimming World® (Alfreton, UK) had access to the study data or were involved in data collection or analyses. DB was supported by the National Institute for Health Research (NIHR) Collaboration for Leadership in Applied Health Research and Care South London (NIHR CLAHRC South London) at King's College Hospital NHS Foundation Trust. The other authors declare that they have no competing interests in relation to this study.

Data Sharing Statement:

The data sets generated and/or analysed as part of this study are not publicly available due to them containing information that could compromise research participant privacy/consent but are available from the corresponding author on reasonable request.

References

1. NHS Maternity Statistics 2017-18 https://files.digital.nhs.uk/C3/47466E/hosp-epis-stat-mat-summary-report%202017-18.pdf Last accessed: October 2019
2. Dudenhausen JW 2015 Prepregnancy body weight and gestational weight gain. *Am J Obstet Gynecol* 2015;591-92
3. Leddy MA, Power ML, Schulkin J The Impact of Maternal Obesity on Maternal and Fetal Health. *Rev Obstet Gynecol* 2008;1(4):170-178
4. Hilson JA, Rasmussen KM, Kjolhede CL. Excessive weight gain during pregnancy is associated with earlier termination of breast-feeding among white women. *The Journal of Nutrition* 2006;136(1):140–146.
5. Vinter CA, Jensen DM, Ovesen P, et al. Postpartum weight retention and breastfeeding among obese women from the randomized controlled Lifestyle in Pregnancy (LiP) trial. *Acta Obstet Gynecol Scand* 2014;93(8):794-801
6. Poston L, Harthoorn LF, van der Beek EM: Obesity in pregnancy: implications for the mother and lifelong health of the child. A consensus statement. *Pediatr Res* 2011;69:175–180.
7. Rooney BL, Schauberger CW, Mathiason MA. Impact of perinatal weight change on long-term obesity and obesity-related illnesses. *Obstet Gynecol* 2005;106:1349–56.
8. Hanson M, Godfrey K, Poston L, et al Pre-conception Health. In: Davies SC Annual Report of the Chief Medical Officer, 2014, The health of the 51%: women. London: Department of Health (2015)
9. Poston L. Obesity in pregnancy: could lifestyle interventions work? *BMC Medicine* 2014;12:201

For peer review only - http://bmjopen.bmj.com/site/about/guidelines.xhtml

10. Amorim Adegboye AR, Linne YM. Diet or exercise, or both, for weight reduction in women after childbirth. *Cochrane Database of Systematic Reviews* 2013, Issue 7. Art. No.: CD005627. DOI: 10.1002/14651858.CD005627.pub3.
11. van der Pligt P, Willcox J, Hesketh KD, et al. Systematic review of lifestyle interventions to limit postpartum weight retention: implications for future opportunities to prevent maternal overweight and obesity following childbirth. *Obesity Reviews* 2013;14:792–805 doi: 10.1111/obr.12053
12. Jolly K, Lewis A, Beach J, et al Comparison of range of commercial or primary care led weight reduction programmes with minimal intervention control for weight loss in obesity: Lighten Up randomised controlled trial. *BMJ* 2011;343:d6500 doi: 10.1136/bmj.d6500
13. Michie S, van Strelen M, West R. The behaviour change wheel: A new method for characterising and designing behaviour change interventions. *Implementation Science* 2011;6:42. doi: 10 1186/1748-5908-6-42.
14. Gidlow C, Povey R, Ellis N, et al Uptake and retention in group-based weight management services. Literature review and behavioural analysis. Final report 2018. Public Health England: London. 2018.
15. Bick D, Taylor C, Bhavnani V, Healey A, Seed P, Roberts S et al (2020) Lifestyle information and commercial weight management groups to support maternal postnatal weight management and positive lifestyle behaviour: the SWAN feasibility randomised controlled trial. BJOG DOI: 10.1111/1471-0528.16043
16. Bandura, A *Social foundations of thought and action: A social cognitive theory*. Prentice-Hall, Inc. 1986.
17. Elfhag K, Rössner S: Who succeeds in maintaining weight loss? A conceptual review of factors associated with weight loss maintenance and weight regain. *Obes Rev* 2005;6(1):67–85.
18. Wing RR, Jeffery RW: Benefits of recruiting participants with friends and increasing social support for weight loss and maintenance. *J Consult Clin Psychol* 1999;67(1):132–138
19. Stubbs RJ, Morris L, Pallister C, et al Weight outcomes audit in 1.3 million adults during their first 3 months' attendance in a commercial weight management programme *BMC Public Health* 2015;15:882
20. O'Cathain A, Hoddinott P, Lewin S, et al. Maximising the impact of qualitative research in feasibility studies for randomised controlled trials: guidance for researchers *Pilot and Feasibility Studies* 2015;1:32
21. Ritchie J, Lewis J: Qualitative research practice: a guide for social science students and researchers. London: Sage 2003
22. Poston L, Bell R, Croker H, et al on behalf of the UPBEAT Trial Consortium. Effect of a behavioural intervention in obese pregnant women (the UPBEAT study): a multicentre, randomised controlled trial. *Lancet Diabetes Endocrinol* 2015;3(10):767–777
23. Davis, DL, Raymond, JE, Clements, V, et al Addressing obesity in pregnancy: The design and feasibility of an innovative intervention in NSW, Australia. *Women Birth* 2012;25:174– 80.
24. Knight, BA, Wyatt, K. Barriers encountered when recruiting obese pregnant women to a dietary intervention. *Nursing Times*. 2010;106:20– 2
25. Blane DN, McLoone P,Morrison D, *et al*. Patient and practice characteristics predicting attendance and completion at a specialist weight management service in the UK: a cross sectional study. *BMJ Open* 2017;7:e018286. doi:10.1136/bmjopen-2017-018286
26. Holton S, East C, Fisher J. Weight management during pregnancy: a qualitative study of women's and care providers' experiences and perspectives *BMC Pregnancy and Childbirth* 2017;17:351

For peer review only - http://bmjopen.bmj.com/site/about/guidelines.xhtml

27. Olander EK, Atkinson L, Edmunds JK, et al The views of pre- and post-natal women and health professionals regarding gestational weight gain: An exploratory study *Sexual & Reproductive Healthcare* 2011;2:43–48
28. Flannery C, McHugh S, Kenny LC, *et al*. Exploring obstetricians', midwives' and general practitioners' approach to weight management in pregnant women with a BMI ≥25 kg/m2: a qualitative study. *BMJ Open* 2019;9:e024808. doi:10.1136/bmjopen-2018-024808
29. Ahern AL, Wheeler GM, Aveyard P, et al Extended and standard duration weight-loss programme referrals for adults in primary care (WRAP): a randomised controlled trial *Lancet* 2017;389:2214–25
30. Craig LE, McInnes E, Taylor N, Grimley R, Cadilhac DA, Considine J, Middleton S. Identifying the barriers and enablers for a triage, treatment, and transfer clinical intervention to manage acute stroke patients in the emergency department: a systematic review using the theoretical domains framework (TDF). Implement Sci. 2016;11:157.
31. Graham-Rowe E, Lorencatto F, Lawrenson J, Burr J, Grimshaw J, Ivers N, Presseau J, Vale L, Peto T, Bunce C. Barriers to and enablers of diabetic retinopathy screening attendance: a systematic review of published and grey literature. Diabet Med. 2018;35:1308-19.
32. Heslehurst N, Newham J, Maniatopoulos G, Fleetwood C, Robalino S, Rankin J. Implementation of pregnancy weight management and obesity guidelines: a meta-synthesis of healthcare professionals' barriers and facilitators using the Theoretical D omains F ramework. Obes Rev. 2014;15:462-86.
33. McDonagh LK, Saunders JM, Cassell J, Curtis T, Bastaki H, Hartney T, Rait G (2018) Application of the COM-B model to barriers and facilitators to chlamydia testing in general practice for young people and primary care practitioners: a systematic review. Implementation Science, 13:130. https://doi.org/10.1186/s13012-018-0821-y

For peer review only

For peer review only - http://bmjopen.bmj.com/site/about/guidelines.xhtml

Table 1: Characteristics of study participants

Characteristics	Intervention group n=98	Survey response n=83*	Interview 6 months n=13	Interview 12 months n=9
**Ethnicity**				
White	49 (50%)	43 (52%)	10 (77%)	6 (67%)
Black	35 (36%)	27 (33%)	3 (23%)	3 (33%)
Asian	8 (8%)	8 (10%)	0 (0%)	0 (0%)
Other	6 (6%)	3 (4%)	0 (0%)	0 (0%)
Unknown	0 (0%)	2 (2%)	0 (0%)	0 (0%)
**Age**				
20-24	7 (7%)	4 (5%)	1 (8%)	0 (0%)
25-29	19 (19%)	17 (20%)	3 (23%)	1 (11%)
30-34	33 (34%)	26 (31%)	4 (31%)	3 (33%)
35-39	33 (34%)	30 (36%)	4 (31%)	4 (44%)
40-44	6 (6%)	6 (7%)	1 (8%)	1 (11%)
**BMI at booking**				
<25 with EGWG**	3 (3%)	3 (4%)	0 (0%)	1 (11%)
25-29.9	55 (56%)	51 (61%)	9 (69%)	6 (67%)
>=30	40 (41%)	29 (35%)	4 (31%)	2 (22%)
**Parity**				
Primigravida	50 (51%)	43 (52%)	9 (69%)	5 (56%)
Multigravida	46(47%)	38 (46%)	3 (23%)	4 (44%)
Unknown	2 (2%)	2 (2%)	1 (8%)	0 (0%)
**Number of sessions attended (data from SW)**				
10+	19 (19%)	19 (23%)	6 (46%)	4 (44%)
6 to 9	8 (8%)	8 (10%)	3 (23%)	1 (11%)
1 to 5	19 (19%)	15 (18%)	3 (23%)	3 (33%)
None	52 (53%)	41 (49%)	1 (8%)	1 (11%)

*includes those who responded either to six month questionnaire only, or to both six and 12 month questionnaires

** EGWG Excessive Gestational Weight Gain

For peer review only

Table 2: Barriers to uptake and retention mapped to COM-B and to potential behaviour change techniques

COM-B	Theme	10+ Slimming World sessions	<10 Slimming World sessions	Behaviour change techniques that could help mitigate barriers
Motivation	weight loss aspirations	• self-perceived as overweight<br>• understood why gained weight<br>• desired to lose weight after birth	• some acknowledged overweight and understood why but viewed weight management as less of a priority<br>• Others did not self-perceive as overweight	Provide information (leaflet/brief consultation) at outset to help influence knowledge and understanding regarding weight loss and consequences of action/no action.<br>• Identity (Framing/reframing; Incompatible beliefs; identity associated with changed behaviour)<br>• Comparison of outcomes (Pros and cons; credible source; comparative imagining of future outcomes)<br>• Goal setting (problem solving)<br>• Natural consequences (Information about health consequences; Salience of consequences; Information about social and environmental consequences
Capability/ Motivation	beliefs and expectations about SW	• SW and resulting weight loss helped change any pre-	• Mistrustful of SW, in part as perceived as failing to prioritise exercise in line with	Leaflet/brief consultation (as above) to include:<br>• Shaping knowledge (Information about antecedents e.g. provide reliable info regarding weight gain/loss;

		existing ideas about how to lose weight, esp re: role of exercise	pre-existing beliefs, seen as unsuitable for breastfeeding women, or felt gap in care e.g in relation to postnatal mental wellbeing	Re-attribution e.g. elicit perceived causes of weight gain/how to lose weight and suggest alternatives)<br>• Goals and Planning (goal setting: behaviour e.g. to make clear at the outset that the SW programme considers the role of exercise but focusses on diet; that 10+ sessions are needed for it to be most effective; Goal setting: outcome – make clear at the outset that the outcome goals are focussed on weight management, but that weight loss may have other positive outcomes such as improved sense of wellbeing/mental health.<br>• Comparison of Outcomes (Pros and cons: discuss pros and cons of exercise vs diet for weight loss)
Capability	understanding and implementing SW	• Understood dietary plan, found easy to follow, planned meals and used online resources.<br>• Considered plan unrestrictive, sustainable and compatible with	• Did not appear to understand the dietary plan, only partial implementation, and experienced difficulties making dietary adjustments.	Development of a bespoke booklet to give to women at randomisation that summarizes key elements of the programme (e.g. some top tips/swaps; a sample 7 day menu plan); as well as optimising the following within the programme:<br>Goals and Planning (goal setting: behaviour; action planning)<br>Feedback & Monitoring (self-monitoring of behaviour e.g. complete food diary and get feedback from consultant)<br>Shaping Knowledge (instruction on how to perform a behaviour)<br>Repetition/Substitution (behaviour practice and

		postnatal lifestyle<br>• Positive benefits for them and their families		rehearsal; graded tasks)
Opportunity	Social context/accessibility	• Identified with group members/social bonds<br>• Positive relationships with group consultants, personalised support<br>• Supported by partners, childcare/sharing SW meals.	• Didn't bond/socially identify with group<br>• Consultants unsympathetic to postnatal challenges<br>• Partner lack of support/partial adaptation to previous meals.	In the leaflet/brief consultation provided at randomisation, women can be reminded that they can change groups and consultants if desired; incorporate anticipation of impact of the programme on the family/partner into brief consultation (e.g. how to discuss with partner; a section in the leaflet for partner/family to read).<br>Social support (unspecified; practical; emotional)<br>Reward and Threat (social incentive: explore whether their family/peers will congratulate them if they lose weight/attend SW sessions; restructure the social environment e.g. by involving those who will be supportive of them attending SW, of dietary changes and weight loss)

For peer review only - http://bmjopen.bmj.com/site/about/guidelines.xhtml

COREQ (COnsolidated criteria for REporting Qualitative research) Checklist

A checklist of items that should be included in reports of qualitative research. You must report the page number in your manuscript where you consider each of the items listed in this checklist. If you have not included this information, either revise your manuscript accordingly before submitting or note N/A.

Topic	Item No.	Guide Questions/Description	Reported on Page No.
**Domain 1: Research team and reflexivity**			
Personal characteristics			
Interviewer/facilitator	1	Which author/s conducted the interview or focus group?	5
Credentials	2	What were the researcher's credentials? E.g. PhD, MD	5
Occupation	3	What was their occupation at the time of the study?	1
Gender	4	Was the researcher male or female?	5
Experience and training	5	What experience or training did the researcher have?	5
Relationship with participants			
Relationship established	6	Was a relationship established prior to study commencement?	5
Participant knowledge of the interviewer	7	What did the participants know about the researcher? e.g. personal goals, reasons for doing the research	5
Interviewer characteristics	8	What characteristics were reported about the inter viewer/facilitator? e.g. Bias, assumptions, reasons and interests in the research topic	5
**Domain 2: Study design**			
Theoretical framework			
Methodological orientation and Theory	9	What methodological orientation was stated to underpin the study? e.g. grounded theory, discourse analysis, ethnography, phenomenology, content analysis	6
Participant selection			
Sampling	10	How were participants selected? e.g. purposive, convenience, consecutive, snowball	5
Method of approach	11	How were participants approached? e.g. face-to-face, telephone, mail, email	5
Sample size	12	How many participants were in the study?	7
Non-participation	13	How many people refused to participate or dropped out? Reasons?	5
Setting			
Setting of data collection	14	Where was the data collected? e.g. home, clinic, workplace	5
Presence of non-participants	15	Was anyone else present besides the participants and researchers?	5
Description of sample	16	What are the important characteristics of the sample? e.g. demographic data, date	17
Data collection			
Interview guide	17	Were questions, prompts, guides provided by the authors? Was it pilot tested?	5
Repeat interviews	18	Were repeat inter views carried out? If yes, how many?	n/a
Audio/visual recording	19	Did the research use audio or visual recording to collect the data?	6
Field notes	20	Were field notes made during and/or after the inter view or focus group?	no
Duration	21	What was the duration of the inter views or focus group?	6
Data saturation	22	Was data saturation discussed?	5
Transcripts returned	23	Were transcripts returned to participants for comment and/or	no

For peer review only

Topic	Item No.	Guide Questions/Description	Reported on Page No.
		correction?	
**Domain 3: analysis and findings**			
Data analysis			
Number of data coders	24	How many data coders coded the data?	6
Description of the coding tree	25	Did authors provide a description of the coding tree?	6
Derivation of themes	26	Were themes identified in advance or derived from the data?	6
Software	27	What software, if applicable, was used to manage the data?	n/a
Participant checking	28	Did participants provide feedback on the findings?	no
Reporting			
Quotations presented	29	Were participant quotations presented to illustrate the themes/findings? Was each quotation identified? e.g. participant number	7-11
Data and findings consistent	30	Was there consistency between the data presented and the findings?	yes
Clarity of major themes	31	Were major themes clearly presented in the findings?	7
Clarity of minor themes	32	Is there a description of diverse cases or discussion of minor themes?	7

Developed from: Tong A, Sainsbury P, Craig J. Consolidated criteria for reporting qualitative research (COREQ): a 32-item checklist for interviews and focus groups. *International Journal for Quality in Health Care*. 2007. Volume 19, Number 6: pp. 349 – 357

Once you have completed this checklist, please save a copy and upload it as part of your submission. DO NOT include this checklist as part of the main manuscript document. It must be uploaded as a separate file.

For peer review only
